# EP300/CREBBP acetyltransferase inhibition limits steroid receptor and FOXA1 signaling in prostate cancer cells

**DOI:** 10.1007/s00018-024-05209-z

**Published:** 2024-04-02

**Authors:** Jasmin Huttunen, Niina Aaltonen, Laura Helminen, Kirsi Rilla, Ville Paakinaho

**Affiliations:** Institute of Biomedicine, University of Eastern Finland, Kuopio, Finland

**Keywords:** Glucocorticoid receptor, Androgen receptor, FOXA1, Chromatin, EP300

## Abstract

The androgen receptor (AR) is a primary target for treating prostate cancer (PCa), forming the bedrock of its clinical management. Despite their efficacy, resistance often hampers AR-targeted therapies, necessitating new strategies against therapy-resistant PCa. These resistances involve various mechanisms, including AR splice variant overexpression and altered activities of transcription factors like the glucocorticoid receptor (GR) and FOXA1. These factors rely on common coregulators, such as EP300/CREBBP, suggesting a rationale for coregulator-targeted therapies. Our study explores EP300/CREBBP acetyltransferase inhibition’s impact on steroid receptor and FOXA1 signaling in PCa cells using genome-wide techniques. Results reveal that EP300/CREBBP inhibition significantly disrupts the AR-regulated transcriptome and receptor chromatin binding by reducing the AR-gene expression. Similarly, GR’s regulated transcriptome and receptor binding were hindered, not linked to reduced GR expression but to diminished FOXA1 chromatin binding, restricting GR signaling. Overall, our findings highlight how EP300/CREBBP inhibition distinctively curtails oncogenic transcription factors’ signaling, suggesting the potential of coregulatory-targeted therapies in PCa.

**Supplementary Information:**

The online version contains supplementary material available at 10.1007/s00018-024-05209-z.

## Introduction

Prostate cancer (PCa) is the most prevalent cancer in men in the Western world, and its primary treatment involves androgen deprivation therapy (ADT) and blockade of androgen receptor (AR) signaling [1, 2]. The AR blockade encompasses the use of anti-androgens like enzalutamide (ENZ), which competes with androgens for binding to AR, and androgen synthesis blockers like abiraterone, which reduce circulating androgen levels. While these therapies initially show efficacy, PCa can adapt during treatment, leading to the development of castration resistant PCa (CRPC) [3, 4]. Unfortunately, patients with CRPC have limited treatment options and a poor survival rate. A hallmark of CRPC is sustained AR signaling, which can occur through multiple mechanisms, including receptor mutations [5], splice variants [6] and the replacement of AR signaling by the glucocorticoid receptor (GR) [1, 7]. Therefore, there is a critical need for new therapeutic approaches to target sustained AR signaling in PCa. This is particularly relevant for GR signaling in ENZ-resistant PCa, as glucocorticoids are commonly administered to PCa patients to alleviate therapy-related side effects and reduce inflammation [8]. However, antagonizing GR signaling does not improve the survival of PCa patients [9].

Steroid receptors, such as AR and GR, are part of the nuclear receptor superfamily and function as hormone-controlled transcription factors (TFs) [1, 10]. Often, but not always, in the absence of a ligand AR and GR reside in the cytoplasm. In contrast type II nuclear receptors, such as retinoic acid and thyroid hormone receptors, are predominantly nuclear regardless of ligand availability [11]. When a ligand binds to the steroid receptor, it triggers the receptor’s movement into the nucleus, where it interacts with chromatin to regulate gene expression. This process involves the receptor-mediated recruitment of various coregulators with histone-modifying and chromatin remodeling activities. These coregulators are commonly shared by both AR and GR [12]. Besides the TFs themselves, dysregulation of coregulator activity can also play a role in the development and progression of cancers, including PCa [13, 14]. As a result, targeting coregulators is considered an important option for cancer therapies [15–17].

One well-known transcriptional coregulator is the E1A binding protein p300 and its paralog, CREB-binding protein (CBP) [18, 19], both of which play significant roles in the progression of PCa and response to endocrine therapy [20, 21]. For consistency, we will use the gene names of these coregulators, EP300, and CREBBP, throughout the text. EP300/CREBBP are histone acetyltransferase (HAT)-containing proteins that acetylate amino acid residues of their target proteins. By acetylating lysine residues of histones, EP300/CREBBP can loosen the interaction between negatively charged DNA and positively charged histones, creating open and accessible chromatin for the binding of TFs and RNA polymerase [22]. In addition to the acetylation “writer” HAT domain, EP300/CREBBP also contains the acetylation “reader” bromodomain (BRD), which the coregulator uses to bind to acetylated proteins [23, 24]. Recent results indicate a clear directionality in how EP300/CREBBP utilizes these domains to “write” and “read” acetylation [25]. Consequently, both domains are essential for the coregulator’s function and represent attractive targets for small-molecule inhibitors [26]. Interestingly, the inhibition of the acetyltransferase function more closely resembles the knockdown of EP300/CREBBP than the inhibition of the BRD function [24].

The potential benefits of targeting EP300/CREBBP in PCa are primarily attributed to its complex formation with AR [27]. Here, cryo-EM investigations showed structural interaction between EP300 with that of the AR-dimer. Consequently, the development of new small-molecule EP300/CREBBP inhibitors has been focused on PCa and AR signaling [16, 17]. A485, an acetyltransferase inhibitor, has demonstrated its ability to restrict AR transcriptional programs and inhibit CRPC tumor growth [16]. On the other hand, CCS1477, a BRD inhibitor, effectively downregulates androgen response and inhibits PCa cell proliferation [17]. However, the impact of these inhibitors on AR chromatin binding has not been extensively investigated. In addition to their effect on AR, studies conducted by us and others indicate that ENZ-resistant PCa cells are more sensitive to EP300/CREBBP inhibition compared to ENZ-naïve PCa cells [28, 29]. Given the significance of GR activity in ENZ-resistant PCa cells [1, 7] and the positive association between EP300 chromatin occupancy in lung cancer cells and GR binding [30], EP300/CREBBP inhibitors could potentially counteract the GR-mediated replacement of AR signaling. Based on our previous data, inhibiting EP300/CREBBP can be employed to modulate GR-mediated transcriptional regulation in ENZ-treated PCa cells [29].

In our current study, we aimed to investigate the impact of EP300/CREBBP acetyltransferase inhibition on the genome-wide actions of AR and GR signaling in PCa cells. To achieve this, we utilized several deep sequencing techniques, including ChIP-seq, ATAC-seq, and RNA-seq. Through our investigation, we made significant discoveries. We found that the EP300/CREBBP acetyltransferase inhibition greatly impairs the AR-regulated transcriptome and hampers receptor chromatin binding. This effect was primarily attributed to the downregulation of *AR*-gene. Similarly, EP300/CREBBP acetyltransferase inhibition also led to the inhibition of the GR-regulated transcriptome and receptor chromatin binding. However, unlike in the case of AR, this inhibition was not due to the downregulation of GR-gene expression. Instead, we observed a substantial loss of FOXA1 chromatin binding upon EP300/CREBBP acetyltransferase inhibition, which subsequently restricted GR signaling. Our results demonstrate that EP300/CREBBP acetyltransferase inhibition not only limits steroid receptor signaling but also drastically curbs FOXA1’s action. These findings provide valuable insights into the complex regulatory mechanisms involving EP300/CREBBP and its impact on steroid receptor and FOXA1 functions in PCa cells.

## Materials and methods

### Cell culture and treatments

22Rv1 (ATCC, #CRL-2505, RRID:CVCL_1045) cells were cultured in RPMI-1640 medium (Gibco, #1187509) supplemented with 10% FBS (Gibco, #11573397), 1 U/µl penicillin, 1 µg/ml streptomycin (Gibco, #15,140,122) and 2 mM L-glutamine (Gibco, #A2916801). VCaP (ATCC, #CRL-2876, RRID:CVCL_2235) cells were cultured in DMEM (Gibco, #41965–039) supplemented with 10% FBS (Cytiva, #11531831) and 1 U/µl penicillin and 1 µg/ml streptomycin (Gibco, #15140122). Both cell lines were maintained at 37 ℃ in 5% CO_2_ conditions. Enzalutamide-treated cells were generated by culturing the cells in growth medium supplemented with 10 µM enzalutamide (ENZ) (MedChemExpress, # HY-70002) for at least 21 days before the start of the experiments passaging or changing the media every 3–4 days. In general, the ENZ treatment time ranged between 21 and 24 days, being at least 3 weeks. These cells are indicated as ENZ-treated in the text and Figures. For the experiments, growth medium was changed to experiment medium supplemented with 5% charcoal-stripped FBS (Gibco, #10270106) for at least one day before the start of treatments. Cells were treated with 1 µM or 10 µM A-485 (A485 for simplicity) (Tocris, #6387), C646 (Sigma, #SML0002), I-CBP112 (Sigma, #SML1134), SGC-CBP30 (Sigma, #SML1133) or CCS1477 (MedChemExpress, #HY-111784). The concentration of A485 used in RNA-seq, ChIP-seq, and ATAC-seq experiments was chosen based on cell proliferation assay and our previous investigation [29]. For hormone treatments, 100 nM dexamethasone (Dex) (Sigma-Aldrich, #D4902) and 100 nM 5α-dihydrotestosterone (DHT) (Steraloids, #A2570-000) was used. The concentration of Dex and DHT used was chosen to ensure the full activation of the steroid receptors. Both AR and GR were predominantly cytoplasmic without cognate steroid hormone treatment irrespective of A485 treatment (Supplementary Fig. S1a-b). The cells were regularly tested to be free of mycoplasma contamination.

### Antibodies

Primary antibodies: anti-GR (Cell Signaling Technology, #12,041, RRID:AB_2631286), anti-AR (custom polyclonal rabbit antiserum) [31], anti-FOXA1 (Abcam, #ab23738, RRID:AB_2104842), anti-EP300 (Santa Cruz Biotechnology, #sc-585, RRID:AB_2231120), anti-H3K27ac (Active Motif, #39,133, RRID:AB_2561016), anti-α tubulin (Santa Cruz Biotechnology, #sc-5286, RRID:AB_628411), anti-H3 (Abcam, #ab1791, RRID:AB_302613). Anti-AR recognize both full length AR and AR-V7 [29]. Secondary antibodies: goat anti-rabbit (Invitrogen, #G-21234), goat anti-mouse (Zymed Laboratories, #81–6520).

### Immunoblotting

Protein isolation for western blot was done as previously described [32]. The following primary antibodies were utilized: anti-GR (1:1000), anti-AR (1:10,000), anti-FOXA1 (1:5000), and anti-H3K27ac (1:500). Anti-tubulin (TUB) (1:3000) and anti-H3 (1:1000) antibodies were used as a control for sample loading. Proteins were detected using Pierce ECL Western Blotting Substrate kit (ThermoScientific, #32,106) and ChemiDoc Imager (Bio-Rad). Protein level quantification was calculated using ImageJ (National Institutes of Health, RRID:SCR_003070). AR, GR, and FOXA1 samples were first normalized to the TUB loading control, and H3K27ac samples to the H3 loading control. Subsequently, all samples were further normalized to the control DMSO treatment.

### Cell proliferation (MTS) assay

For cell proliferation assay, the 22Rv1 and VCaP cells were seeded onto 96-well plates (22Rv1, 10 000 cells per well; VCaP, 30 000 cells per well). Plain medium wells were used to extract background. The start of the treatments was determined as day 0 and cell proliferation was assessed at day 4 using colorimetric CellTiter 96 Aqueous One Solution Cell Proliferation Assay (MTS) kit (Promega, #G3580) according to manufacturer’s protocol. The absorbance was measured at 492 nm wavelength using Multiskan EX plate reader (Thermo Scientific). Results were normalized to day-0 and shown as fold change.

### RNA-seq and data analysis

For RNA-sequencing (RNA-seq), the cells were seeded onto 12-well plates (22Rv1, 200,000 cells per well; VCaP, 400,000 cells per well). Medium was changed into charcoal stripped medium (with or without ENZ) two days before the inhibitor treatment. The cells were treated with 1 µM A485 or DMSO for 24 h and with vehicle or steroid hormone (100 nM Dex or 100 nM DHT) 18 h prior the RNA extraction. Hence, the vehicle or steroid hormone was added to the cells 6 h after adding A485 or DMSO. Total RNA was extracted using Monarch Total RNA MiniPrep kit (New England BioLabs, #T2010S). Quality and quantity of the isolated RNA were determined with NanoDrop One (Thermo Scientific) and with Agilent 2100 Bioanalyzer using RNA 6000 Nano kit (5067–1511). All samples had RIN > 9, indicating high integrity of RNA. RNA-seq libraries were generated using NEBNext Poly(A) mRNA Magnetic Isolation Module (New England BioLabs, #E7490) and NEBNext Ultra II Directional RNA Library Prep Kit (New England BioLabs, #E7765) according to manufacturer’s protocol. Library quality was assessed with Agilent 2100 Bioanalyzer using DNA 1000 Analysis kit (Agilent, #5067–1504). Two biological replicate samples were sequenced with Illumina NextSeq 500 (75SE) or Illumina Nextseq 2000 (100SE). Principal component analysis (PCA) indicated clear separation of treatments from each other (Supplementary Fig. S2a). Since RNA-seq was performed with two replicate samples, some of the biological effects could elute our analyses.

RNA-seq data was aligned to hg38 genome using STAR2.7 [33] (RRID:SCR_004463) with default settings and max 10 mismatches and max 10 multi-mapped reads. Differentially expressed genes were then analyzed with DESeq2 [34] (RRID:SCR_015687) through HOMER [35] (RRID:SCR_010881) for all comparisons. Total count per gene was calculated using transcripts per million (TPM) normalization and other than protein coding genes were filtered out as outliers. Genes with TPM > 0.5 at least in one sample in any treatment were considered as expressed. Differentially expressed genes were then defined as FDR < 0.05 and log2 fold change ± 0.5 between differently treated samples. All RNA-seq data values are presented in Supplementary Table S1. Volcano plot of log2 fold change and -log10 adjusted p-value of indicated treatments was used to display A485-mediated change of expressed protein-coding genes. Differentially expressed gene clusters were used in Metascape [36] pathway analysis using the Hallmark Gene Sets with criteria p < 0.01, at least three gene overlap per gene set, and > 1.5 pathway enrichment. Pathway enrichment data is presented in Supplementary Table S2. Prostate cancer patient data was obtained from cBioPortal [37] (RRID:SCR_014555), PCaDB [38], or Prostate Cancer Atlas [39]. The Cancer Cell Line Encyclopedia (CCLE) proteomics data was obtained from supplemental material [40]. Public RNA-seq and proteomics data used is shown in Supplementary Table S3.

### ChIP-seq

Chromatin immunoprecipitation (ChIP) was done as previously described [41]. 22Rv1 and VCaP (with or without ENZ) cells were plated onto 10-cm plates (22Rv1, 3 million cells per plate; 22Rv1-ENZ, 4 million cells per plate; VCaP, 10 million cells per plate; VCaP-ENZ, 11 million cells per plate) and grown for 72 h before changing to stripped medium (as above with RNA) for 24 h. For the experiments, cells were treated with 1 µM A485 or DMSO for 24 h and 100 nM Dex or 100 nM DHT for the last 1 h before the start of ChIP. Hence, the vehicle or steroid hormone was added to the cells 23 h after adding A485 or DMSO. In EP300 ChIP-seq, the cells were treated with 100 nM Dex or EtOH for 1 h. For ChIP, chromatin was fragmented to an average size of 150–300 bp by sonication (Diagenode, #UCD-300). Antibodies were coupled to magnetic protein G Dynabeads (Invitrogen, 10004D) for at least 6 h, and sonicated lysates were incubated with antibody-coupled beads for o/n at 4 °C. Antibodies used per IP: GR, 12.5 µl; AR, 3 µl; FOXA1, 2 µg; H3K27ac, 2 µg; EP300, 2 µg. Two IP samples were pooled for one ChIP-seq sample. Sequencing libraries were generated using NEBNext Ultra II DNA Library Prep Kit (New England BioLabs, #E7645L) according to manufacturer’s protocol. Analysis of library quality was done with Agilent 2100 Bioanalyzer using DNA 1000 Analysis kit (Agilent, #5067–1504). At least two biological replicate samples were sequenced with Illumina NextSeq 500 (75SE) or Illumina Nextseq 2000 (100SE). The number of biological replicate samples followed the ENCODE Consortium criteria.

### ATAC-seq

For assay for transposase-accessible chromatin with sequencing (ATAC-seq), 22Rv1 and VCaP (with or without ENZ) cells were plated to 10 cm plates (22Rv1, 2,5 million cells per plate; 22Rv1-ENZ, 3,5 million cells per plate; VCaP, 10 million cells per plate; VCaP-ENZ, 11 million cells per plate) and grown for 96 h before changing to stripped medium for 24 h. Cells were treated with 1 µM A485 or DMSO for 24 h and 100 nM Dex or EtOH for the last hour prior starting ATAC. Hence, the vehicle or steroid hormone was added to the cells 23 h after adding A485 or DMSO. The ATAC-seq was performed as previously described [42, 43]**.** Briefly, cells were detached from the plates using 2 ml of Accutase (Thermo Fisher Scientific). For nuclei isolation, the cell pellets were resuspended in a concentration of 5 million cells per ml in Buffer A [15 mM Tris–HCl (pH 8), 15 mM NaCl, 60 mM KCl, 1 mM EDTA, 0.5 mM EGTA, 0.5 mM spermidine (Sigma-Aldrich, #S2626), 1X protease inhibitor cocktail] and diluted with same amount of Buffer A + 0,04% IGEPAL (Sigma, #I8896) to obtain a concentration of 2.5 million cells per ml with 0.02% (w/v) IGEPAL. After 10 min incubation samples were washed once with Buffer A (without IGEPAL) and two times with ATAC resuspension buffer [10 mM NaCl, 10 mM Tris–HCl, 3 mM MgCl_2_]. Isolation of nuclei was verified by Trypan Blue counting. Tn5 transposition reaction was done using 2.5 µl tagmentase (Diagenode, #C01070012-30). From each sample, 100 000 nuclei were transferred to the reaction. After adding the transposition reaction mix, the samples were incubated 45 min at 37 °C with 800 rpm shaking, and subsequently DNA was purified using Monarch PCR & DNA Cleanup Kit (New England BioLabs, #T1030). Amplification of transposed DNA fragments was done using PCR (Biometra T3 Thermocycler) and qPCR (Roche LightCycler 480 Instrument II) and samples were barcoded using published primers [42]. Amplified fragments were size selected (150–800 bp) using SPRIselect beads (Beckman Coulter, #B23318). Analysis of library quality was done with Agilent 2100 Bioanalyzer using High Sensitivity DNA Analysis kit (Agilent, #5067–4626). Two biological replicate samples were sequenced with Illumina NextSeq 500 (40PE) or Illumina Nextseq 2000 (50PE). The number of biological replicate samples followed the ENCODE Consortium criteria.

### ChIP-seq and ATAC-seq data analysis

For ChIP-seq, read quality filtering and alignment to hg38 genome using Bowtie [44] (RRID:SCR_005476) was performed as previously described [41]. For ATAC-seq, read quality filtering and alignment to hg38 genome using Bowtie2 [45] (RRID:SCR_016368) was performed as previously described [43]. ATAC-seq data was not divided into nucleosome-free and nucleosomal fragments. For all samples, most of the ATAC-seq fragments were below mono-nucleosomal size (Supplementary Table S4). Downstream data analysis was performed using HOMER [35]. Peaks in each dataset were called using findPeaks with style factor, FDR < 0.01, > 25 tags, > fourfold over control sample and local background. ChIP input sample used as a control sample for ChIP-seq and Tn5-treated DNA sample [29] used as a control sample for ATAC-seq. Comparison of peak overlap per condition for each dataset is shown in Supplementary Fig. S2b. Overlap was performed with mergePeaks. getDifferentialPeaks.pl was used to isolate differential binding peaks between conditions (Poisson p-value < 0.0001 and FC > 3 for GR and AR, or FC > 2 for FOXA1 and H3K27ac). The enrichment at unchanged sites (UN) remained the same or the change was less than the required FC. The enrichment at decreased sites (DN) showed complete loss of binding or the decrease was at least the required FC. The enrichment at increased sites (UP) showed appearance of binding or the increase was at least the required FC. For the GR the change was first determined based on ENZ treatment, and subsequently with A485 treatment. For the rest the change was determined based on A485 treatment (Supplementary Fig. S2c). If the peak number for a given UP or DN comparison was small (< 250 peaks), the peaks were excluded from the downstream analyses. The genomic location of the peaks was similar between peak clusters. ChIP-seq peaks are presented in Supplementary Table S4. For determination of the prevalence of H3K27ac signal decrease at ATAC A485-DN sites, collection of 10 000 random ATAC sites were utilized. The random sites were selected from union of ATAC sites from all conditions. To evaluate the effect of A485 on ATAC-seq or H3K27ac ChIP-seq signal at the GR, AR and FOXA1 binding clusters, the sites were divided based on the degree of signal loss. The division was either less than or more than twofold signal loss. The effect of A485 on H3K27ac ChIP-seq signal at ATAC-seq clusters, and the effect of A485 on ATAC-seq signal at H3K27ac ChIP-seq clusters was evaluated in similar fashion. GR binding sites with A485-reduced change for both FOXA1 and GR (GR-FOXA1 A485-DN) were determined based on the change between control and A485 treatment. Log2 fold change (DMSO/A485) for both TFs was required to be over one. For GR-FOXA1 signature, 20 Dex-regulated genes that displayed the most prominent loss of FOXA1 binding after A485 exposure were selected, and patient expression and survival data was obtained from cBioPortal [37]. For each patient the expression of the signature genes was normalized to *NR3C1* expression, and patients were stratified into low and high GR-FOXA1 signature groups based on the median Z-score. GR-FOXA1 signature genes are listed in Supplementary Table S6. Heatmaps were generated with 20 bp bins surrounding ± 1 kb area around the center of the peak. The peaks in the heatmaps were called as a peak with findPeaks in at least one of the conditions. Box plots represented log2 tag counts. All plots were normalized to 10 million mapped reads and further to local tag density, tags per bp per site. De novo motif searches were performed using findMotifsGenome.pl with default settings. The results from the motif searches were displayed as heatmaps representing % of sites with motif. Only motifs that had 20% or higher enrichment in one of the peak clusters were included in the heatmaps. Motif heatmaps were generated using hierarchical clustering with Euclidean distance. Complete motif data are presented in Supplementary Table S5. Gene-to-peak association (gene-centric analysis) was performed using annotatePeaks.pl measuring the linear distance from target gene TSS to center of the peak. The GR binding peaks were categorized into two groups, A485-UN and A485-DN. Differentially expressed genes from both naïve and ENZ-treated samples were combined for the analysis. Public ChIP-seq data used is shown in Supplementary Table S3 [29, 46, 47].

### Confocal microscopy and FRAP analysis

For the Fluorescence Recovery After Photobleaching (FRAP) analysis, 22Rv1 cells were plated onto 8-well chamber slides (Ibidi GmbH, #80826), 45,000 cells per well. The cells were transfected with constructs expressing eGFP-tagged GR or FOXA1 or TBP using Lipofectamine 3000 reagent (Invitrogen, #L3000008) following the manufacturer’s protocol. eGFP-GR contains human GR in EGFP-C2 vector, eGFP-FOXA1 contains mouse FOXA1 in EGFP-C3 vector, and eGFP-TBP contains human TBP in EGFP-C1 vector. The growth medium was replaced with stripped medium 24 h post-transfection, simultaneously with the inhibitor treatment. Transfected cells were treated with 1 µM A485 or DMSO for 24 h and 100 nM Dex for 1 h before the start of imaging. Imaging was conducted within a 3-h window following the Dex treatment. Measurements with eGFP-GR, -FOXA1 and -TBP were performed in individual experiments. To display the minimal nuclear translocation of AR and GR with only A485 treatment, 22Rv1 cells were transfected as above with eGFP-AR or eGFP-GR construct. eGFP-AR contains human AR in EGFP-C2 vector. Transfected cells were treated with 1 µM A485 or DMSO for 24 h and with or without cognate hormone (100 nM DHT or 100 nM Dex) for 1 h before the start of imaging. The fluorescent images were obtained with a Zeiss Axio Observer inverted microscope (40 × NA 1.3 oil objective) equipped with a Zeiss LSM 800 confocal module (Carl Zeiss Microimaging GmbH). For FRAP analysis, bleach pulses were applied with 75% laser intensity in regions of interest (ROIs) sized at 5 μm by 2,5 μm. Sequential images were collected over a 20–60 s time-period. Fluorescence intensity in the ROI was normalized based on background fluorescence and general bleaching measured during the imaging. Data analysis was performed with ZEN 2012 SP1 (black edition) software (Carl Zeiss Microimaging GmbH, RRID:SCR_018163) using a mono-exponential model: I = IE – I1 * exp (– t / T1), where IE represents the initial intensity, I1 is the amplitude of the fitted curve (mobile fraction), t denotes time in seconds, and T1 is the time constant. The half-time of the recovery was calculated using the following formula: -thalf = (ln 0.5) * T1. The number of single cells analyzed is as follows: GR, n = 75; FOXA1, n = 75; TBP, n = 12. Three independent experiments were conducted for eGFP-GR and -FOXA1.

### Statistical analysis

Statistical significance is indicated with the p-value or with asterisks. Single asterisk denotes p-value < 0.05, double asterisks denote p-value < 0.01, and triple asterisks denotes p-value < 0.001. Non-significant p-values are not indicated. Statistical significance was determined using unpaired t-test (two conditions) or One-way ANOVA with Bonferroni post hoc test (three or more conditions). Statistical analyses used for the determination of differentially expressed genes with RNA-seq are indicated in the RNA-seq and data analysis section. Statistical analyses used for the determination of peaks and differential peak clusters with ChIP-seq and ATAC-seq are indicated in the ChIP-seq and ATAC-seq data analysis section. Statistical significance for the patient survival was determined using log-rank test. Correlation of patient mRNA expression levels was determined with Pearson Correlation Coefficiency (PCC) with statistical significance calculated with t-distribution with n-2 degrees of freedom. Statistical significance for the proportion of binding sites with over twofold difference in ATAC-seq or H3K27ac ChIP-seq signal was determined using Chi-squared test with one degrees of freedom. The sample sizes are indicated in the Figure captions. All bar graphs represent mean ± standard deviation (SD). Box plots were generated using Tukey method with interquartile range (IQR) depicting the 25th, 50th and 75th percentile as box with the median as black bar. The whiskers extend 1.5xIQR beyond the box, and outliers are not depicted but are included in the statistical analyses.

## Results

### EP300/CREBBP acetyltransferase inhibition influences cell growth-related pathways in prostate cancer cells

**Fig. 1:**
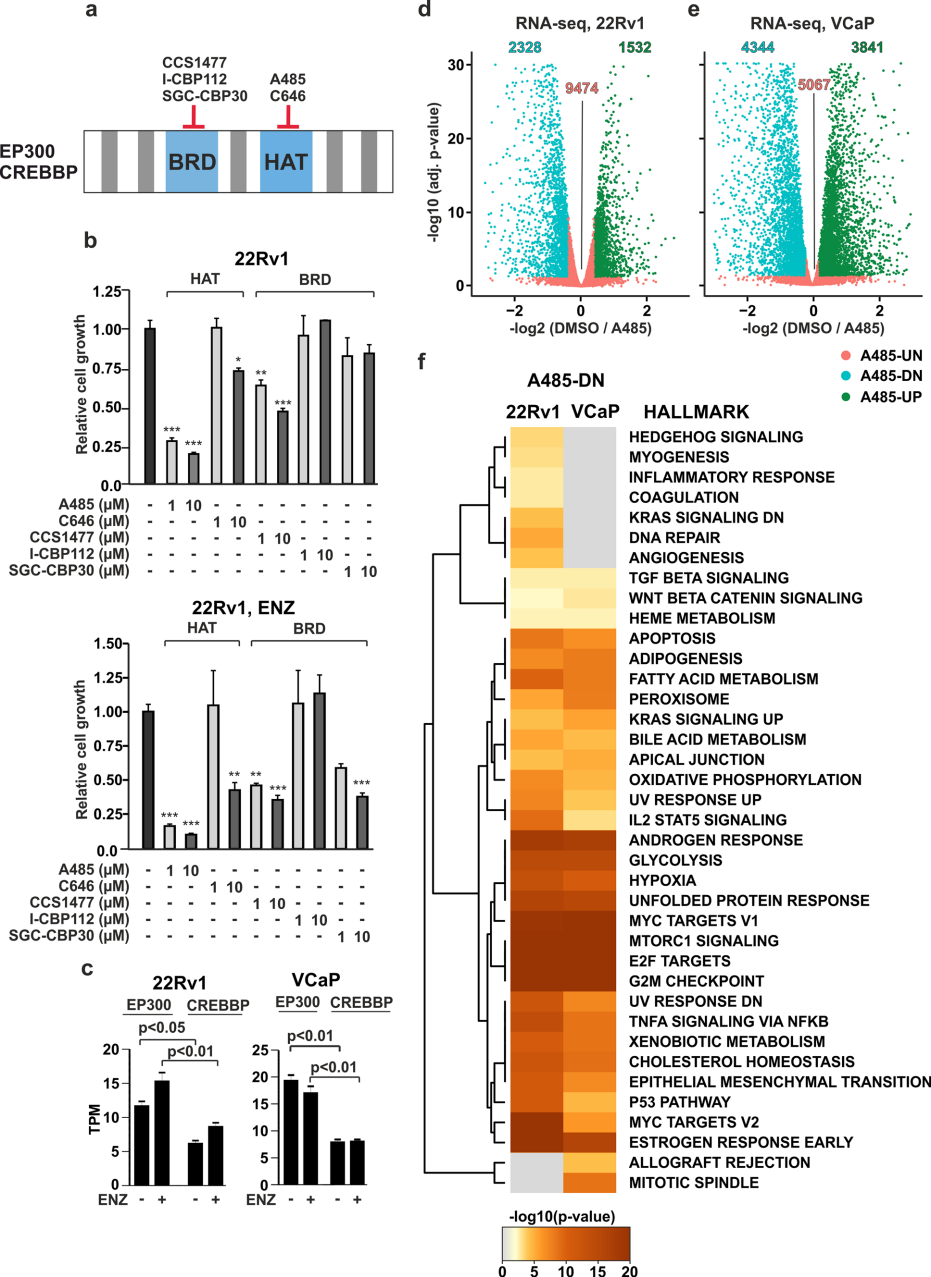
A485 decreases cell proliferation and influences cell growth-related pathways in prostate cancer cells. **a** Schematic representation of the EP300/CREBBP protein domain structure. Highlighted compounds are the acetyltransferase domain (HAT) inhibitors; A485 and C646; and the bromodomain (BRD) inhibitors; CCS1477, I-CBP112, and SGC-CBP30. **b** Bar graphs depict relative cell proliferation of ENZ-naïve (upper) and ENZ-exposed (lower) 22Rv1 cells treated with indicated EP300/CREBBP inhibitors for 96 h. Data is normalized to cell proliferation at the start of the experiment, and represent mean ± SD, n = 4. Statistical significance calculated using One-way ANOVA with Bonferroni post hoc test. *, p < 0.05; **, p < 0.01; ***, p < 0.001. **c** Bar graphs depict *EP300* and *CREBBP* expression levels in ENZ-naïve or ENZ-exposed 22Rv1 (left) and VCaP (right) cells from RNA-seq data. Data shown as transcripts per million (TPM). Statistical significance calculated with One-way ANOVA with Bonferroni post hoc test. n = 2. **d**, **e** Volcano plot of expressed protein coding genes depicting log2 fold change values of A485/DMSO (x-axis) and -log10 adjusted p-value (y-axis) RNA-seq data from **d** 22Rv1 cells or **e** VCaP cells. Number of genes that are unchanged (A485-UN, red), decreased (A485-DN, blue), or increased (A485-UP, green) after A485 treatment (FDR < 0.05, log2 fold change ± 0.5) are indicated in the plot. **f** Hallmark Gene Sets pathway analysis of A485-downregulated (A485-DN) genes from 22Rv1 and VCaP RNA-seq data. Color scale represents -log10 p-value

The significance of both EP300 and CREBBP in PCa has been acknowledged [16, 17]. Interestingly, mRNA expression levels of EP300/CREBBP remain relatively stable throughout PCa progression (Supplementary Fig. S3a) [39]. Furthermore, patient stratification based on EP300/CREBBP expression minimally impacts survival rates (Supplementary Fig. S3b) [3, 48, 49]. Thus, the mere expression of these coregulators does not fully elucidate their importance in PCa. Given that EP300’s TF interaction domains are crucial for its chromatin action [50], the relationship between EP300/CREBBP and the recruiting TF likely contributes to the coregulators significance. Supporting this notion, the mRNA expression of *EP300* and *CREBBP* strongly correlates with the *AR*-gene in PCa patients (Supplementary Fig. S4a) [39], particularly in primary and AR-positive CRPC patients. These findings underscore the potential of targeting these coregulators as a promising strategy to inhibit oncogenic AR signaling in PCa [16, 17]. In our previous work, we demonstrated that inhibiting EP300/CREBBP enzymatic activity restricts the proliferation of PCa cells [29]. Building on this finding, we evaluated various chemical inhibitors targeting the EP300/CREBBP HAT domain (A485, C646) and the BRD (CCS1477, I-CBP112, SGC-CBP30) (Fig. 1a) to assess their impact on PCa cell proliferation. Among the tested compounds, A485 exhibited the highest efficacy in inhibiting cell proliferation in both ENZ-naïve and ENZ-treated 22Rv1 cells (Fig. 1b). We confirmed the cell proliferation effect of A485 and CCS1477 in ENZ-naïve and ENZ-treated VCaP cells (Supplementary Fig. S4b). Based on A485’s prominent effect, we performed RNA-seq in 22Rv1 and VCaP cells exposed to A485 to gain a genome-wide perspective of the gene programs regulated by EP300/CREBBP. At the level of mRNA, both cell lines expressed significantly more EP300 compared to CREBBP (Fig. 1c). However, publicly available quantitative proteomic data indicated slightly higher levels of CREBBP compared to EP300 in both cell lines (Supplementary Fig, S4c). The A485 treatment influenced 29% and 60% of the expressed genes in 22Rv1 and VCaP cells, respectively (Fig. 1d, e). Consistent with the cell proliferation results, the A485-inhibited genes were enriched in pathways related to apoptosis and cell cycle (Fig. 1f). Moreover, androgen response and MYC pathways were also impacted. Interestingly, these pathways were also enriched in 22Rv1 cells exposed to CCS1477 [17], suggesting that inhibiting EP300/CREBBP activity through acetyltransferase or BRD elicits similar responses in PCa cells. Given the prominent effect on androgen response, we utilized A485 in the subsequent analyses of steroid receptor signaling.

### A485 restricts AR chromatin occupancy and transcriptional activity through AR-gene downregulation

**Fig. 2:**
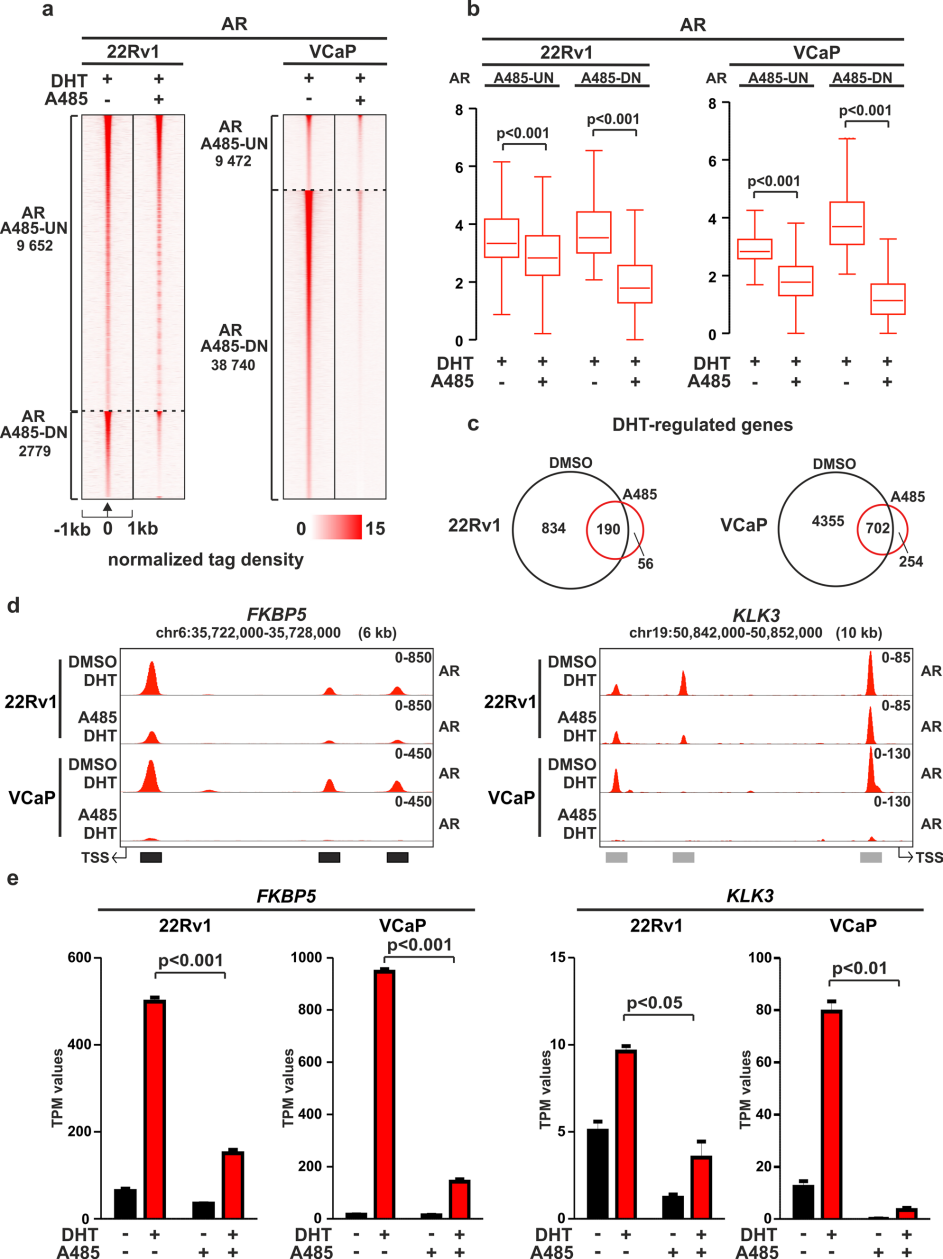
A485 restricts AR chromatin occupancy and transcriptional activity. **a** AR ChIP-seq profiles at AR A485-UN and AR A485-DN sites in 22Rv1 and VCaP cells. AR A485-UN represents unchanged and AR A485-DN decreased ARBs. Heatmaps represent ± 1 kb around the center of the AR peak. Binding intensity (tags per bp per site) scale is noted below on a linear scale. **b** Box plots represent the normalized log2 tag density of AR ChIP-seq at the indicated sites in 22Rv1 and VCaP cells. Statistical significance calculated using One-way ANOVA with Bonferroni post hoc test. All heatmaps and box plots are normalized to a total of 10 million reads. **c** Venn diagrams of DHT-regulated genes irrespective of nearby AR peak location in VCaP and 22Rv1 cells from RNA-seq data before (black circle) and after (red circle) A485 treatment. **d** Genome browser track examples of AR ChIP-seq at *FKBP5* and *KLK3* loci in 22Rv1 and VCaP cells. Black boxes denote intragenic regions, and grey boxes denote intergenic regions. The location of *FKBP5* and *KLK3* transcription start site (TSS) is indicated with arrow. *FKBP5* TSS is located 33 kb and *KLK3* TSS is located 4 kb away from the nearest ARBs. **e** Bar graphs depict *FKBP5* and *KLK3* expression levels in VCaP and 22Rv1 cells from RNA-seq data. Data shown as transcripts per million (TPM). Statistical significance calculated with One-way ANOVA with Bonferroni post hoc test. n = 2

Previously, studies have indicated that A485 does not inhibit AR binding to the PSA enhancer [16], while CCS1477 inhibits AR binding to selected enhancers [17]. However, there is currently a lack of genome-wide knowledge regarding how EP300/CREBBP inhibition affects AR signaling and chromatin binding. To address this, we conducted AR ChIP-seq analyses in 22Rv1 and VCaP cells, revealing that A485 treatment significantly reduced the genome-wide chromatin binding of AR in both cell lines (Fig. 2a, b). The effect was more pronounced in VCaP cells, where 80% of AR binding sites (ARBs) displayed a significant reduction in AR signal (AR A485-DN) (Fig. 2a). In 22Rv1 cells, 22% of ARBs were reduced. The AR A485-DN ARBs showed higher enrichment of androgen response elements (AREs) and lower enrichment of FOXA1 and HOXB13 motifs compared to the unchanged sites (AR A485-UN) (Supplementary Fig. S5a, Supplementary Table S5). Published FOXA1 [29] and HOXB13 ChIP-seq data [47] confirmed that AR A485-DN ARBs exhibited lower chromatin occupancy of these TFs in both 22Rv1 and VCaP cells (Supplementary Fig. S5b-c). This indicates that A485 primarily targets AR chromatin occupancy at its canonical ARE-containing enhancers.

Consistent with the significant loss of AR binding, the DHT-regulated transcriptome was clearly reduced upon A485 treatment in both cell lines (Fig. 2c, Supplementary Table S1). Although the extent of AR binding inhibition upon A485 exposure varied between VCaP and 22Rv1 cells, the number of DHT-regulated genes decreased by over 80% with A485 in both cell lines. Target genes, such as *FKBP5* and *KLK3* (gene encoding PSA), demonstrated both decreased AR binding and reduced DHT-induction (Fig. 2d-e). Interestingly, we observed a substantial decrease in both *AR*-gene transcript levels (Supplementary Fig. S5d) and AR protein levels, including constitutively active variant AR-V7 (Supplementary Fig. S7f), upon A485 treatment. Furthermore, given the strong positive correlation between *AR* and *EP300*/*CREBBP* in PCa patients (Supplementary Fig. S4a), it is likely that the decrease in *AR*-gene expression through inhibition of EP300/CREBBP activity is a key contributor to AR signal restriction.

### Inhibition of EP300/CREBBP disrupts GR binding and reduces chromatin accessibility at receptor binding sites

**Fig. 3:**
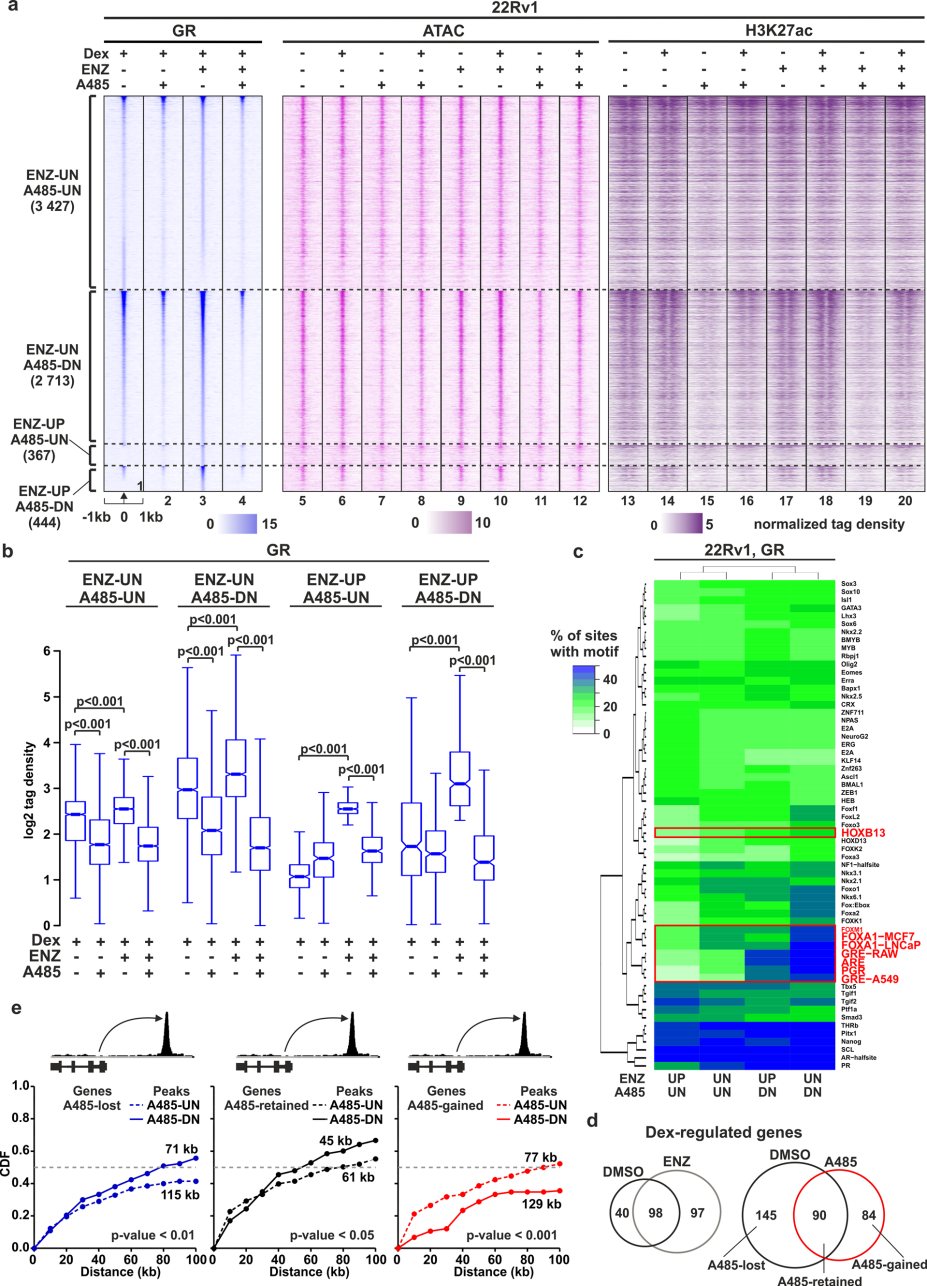
A485 disrupts GR chromatin binding and reduces chromatin accessibility at GR binding sites. **a** GR ChIP-seq, ATAC-seq, and H3K27ac ChIP-seq profiles at ENZ-UN A485-UN, ENZ-UN A485-DN, ENZ-UP A485-UN, and ENZ-UP A485-DN sites in ENZ-naïve and ENZ-exposed 22Rv1 cells. UN represents unchanged, UP represents increased, and DN represents decreased GRBs upon the respective treatments. Each heatmap represents ± 1 kb around the center of the GR peak. Binding intensity (tags per bp per site) scale is noted below on a linear scale. **b** Box plots represent the normalized log2 tag density of GR ChIP-seq at the indicated sites. Statistical significance calculated using One-way ANOVA with Bonferroni post hoc test. All heatmaps and box plots are normalized to a total of 10 million reads. **c** De novo motif enrichment at indicated GRBs in 22Rv1 cells. Enrichment is displayed as a heatmap representing % of sites with motif. The scale is displayed on the side of the heatmap with white–green–blue indicating the prevalence of enrichment. Red rectangle highlights relevant motifs. **d** Venn diagrams of Dex-regulated genes from (left) ENZ-naïve (black circle) and ENZ-exposed (grey circle) 22Rv1 cells, and (right) DMSO (black circle) and A485 (red circle) exposed 22Rv1 cells. **e** Association of Dex-regulated genes and A485-UN/-DN GRBs from 22Rv1 cells. Dex-regulated genes in ENZ-naïve and ENZ-exposed cells were combined. Associated genes were divided to A485-lost (blue line), A485-retained (black line) and A485-gained (red line) sections. A485-UN depicted as dashed line and A485-DN as solid line. Data is represented as cumulative distribution function (CDF). Median depicted as grey dashed line. Statistical significance calculated with One-way ANOVA with Bonferroni post hoc test

In addition to AR, the transcriptional activity of GR is relevant for PCa, as it can mediate antiandrogen-resistance by usurping AR’s oncogenic role [7, 29]. Therefore, we aimed to explore whether GR signaling is impacted by EP300/CREBBP acetyltransferase inhibition and if its mechanisms of action are like that of AR. Analysis of PCa patient data revealed a strong positive correlation between *EP300*/*CREBBP* and *NR3C1* (GR-gene) expression (Supplementary Fig. S4d), particularly in primary, AR-positive, and double-negative CRPC patients. We have previously demonstrated a significant inhibitory effect of EP300/CREBBP inhibition on GR-mediated target gene regulation in 22Rv1 and VCaP cells [29]. However, the genome-wide impact of A485 on GR action remains elusive. To address this, we conducted GR ChIP-seq experiments in both 22Rv1 and VCaP cells. Additionally, since GR predominantly binds to open chromatin sites in PCa cells [29], we also assessed chromatin accessibility using ATAC-seq. Moreover, considering that GR signaling is potentiated with AR repression, we performed the experiments in both ENZ-naïve and ENZ-treated cells. The GR binding sites (GRBs) obtained from GR ChIP-seq were categorized into four peak clusters based on the changes upon ENZ treatment (ENZ-UN or ENZ-UP) and A485 treatment (A485-UN or A485-DN); ENZ-UN/A485-UN, ENZ-UN/A485-DN, ENZ-UP/A485-UN, and ENZ-UP/A485-DN. Notably, A485 significantly reduced GR chromatin binding, impacting approximately 45% of all GRBs in 22Rv1 cells (Fig. 3a, b). The decrease in GR binding was also observed at A485-UN sites when considering only signal density. Interestingly, the loss of GR binding was more pronounced in ENZ-treated compared to ENZ-naïve 22Rv1 cells (Fig. 3a; lane #1–2 vs. #3–4). Similar effects on GR binding were observed in VCaP cells (Supplementary Fig. S6a-b). Furthermore, de novo motif analysis revealed the enrichment of FOXA1 and HOXB13 motifs, along with the GRE motif, at the GR binding clusters (Fig. 3c, Supplementary Fig. S6c, Supplementary Table S5).

Interestingly, ATAC-seq analyses indicated that the decreased GRBs (A485-DN) exhibited significantly reduced chromatin accessibility (Fig. 3a; lane #6 vs. #8, and lane #10 vs. #12) (Supplementary Fig. S6a, S6d and S7a-b). The reduction was more prominent in A485-DN compared to A485-UN sites, suggesting that chromatin accessibility plays a critical role in the EP300/CREBBP inhibition-mediated restriction of GR binding. To verify the role of EP300/CREBBP at these sites, we conducted EP300 and H3K27ac ChIP-seq analyses. The former was used to confirm EP300 binding to GRBs, while the latter served as a proxy for the reduction of EP300 activity upon A485 exposure. The ChIP-seq analyses confirmed that EP300 binds to the GRBs (Supplementary Fig. S6e and S7c), and this binding is significantly enhanced by Dex-treatment, especially in ENZ-treated 22Rv1 cells. Furthermore, the Dex-induced recruitment of EP300 was more prevalent at A485-DN GRBs compared to A485-UN GRBs. Consistent with EP300 binding, A485 treatment resulted in a decreased level of H3K27ac, particularly at the A485-DN GRBs (Fig. 3a; lane #13–14 vs. #15–16, and lane #17–18 vs. #19–20) (Supplementary Fig. S7d-e). H3K27ac protein levels were also reduced by A485 treatment (Supplementary Fig. S7f), and the chromatin H3K27ac occupancy was significantly inhibited at approximately 20% of sites (Supplementary Fig. S7h). However, A485 treatment did not completely remove H3K27ac from chromatin, as previously shown [51, 52]. These results suggest that the inhibition of GR binding upon A485 treatment occurs due to the reduction of H3K27ac and chromatin accessibility.

Given the substantial alterations in GR binding upon A485 treatment, we investigated the impact of EP300/CREBBP acetyltransferase inhibition on GR-mediated transcription with RNA-seq. Consistent with our previous data [29], ENZ treatment led to a clear increase in the number of Dex-regulated genes (Fig. 3d, Supplementary Table S1). Among the total Dex-regulated genes in ENZ-naïve and ENZ-treated 22Rv1 cells, 38% of genes retained their Dex regulation (A485-retained), while 62% of genes lost their Dex-regulation upon A485 exposure (A485-lost). Additionally, there were several genes that gained Dex-regulation with A485 treatment (A485-gained). The union of A485-lost and -gained genes were associated with pathways related to cell growth, development, and differentiation, as well as linked to hormone response, and steroid receptor signaling pathways (Supplementary Table S2). Similar effects were observed in VCaP cells (Supplementary Fig. S6f). Next, we performed an analysis of the association between GRBs and Dex-regulated genes upon EP300/CREBBP acetyltransferase inhibition. We observed a clear correlation between the loss of GR binding and the disappearance of Dex-regulation in 22Rv1 cells (Fig. 3e). A485-lost genes were found to be significantly closer to the A485-DN GRBs compared to the A485-UN GRBs. Furthermore, A485-retained genes were also significantly more closely associated with the A485-DN GRBs. However, A485-gained genes were significantly closer to the A485-UN GRBs rather than the A485-DN GRBs. This suggests that A485-gained genes obtain their regulation through an indirect mechanism. Interestingly, unlike AR-regulation, the *NR3C1* transcript levels and GR protein levels did not predominantly decrease upon A485 exposure (Supplementary Fig. S7f-g). Instead, *NR3C1* expression was significantly increased by both ENZ and A485 treatment. This suggest that EP300/CREBBP acetyltransferase inhibition has largely different mechanism to restrict AR versus GR signaling. Overall, the data suggest that A485 can modulate GR’s transcriptional regulatory capacity through the restriction of chromatin accessibility at GRBs.

### Accessible chromatin sites sensitive to A485 exposure are highly enriched with FOXA1 motifs

**Fig. 4:**
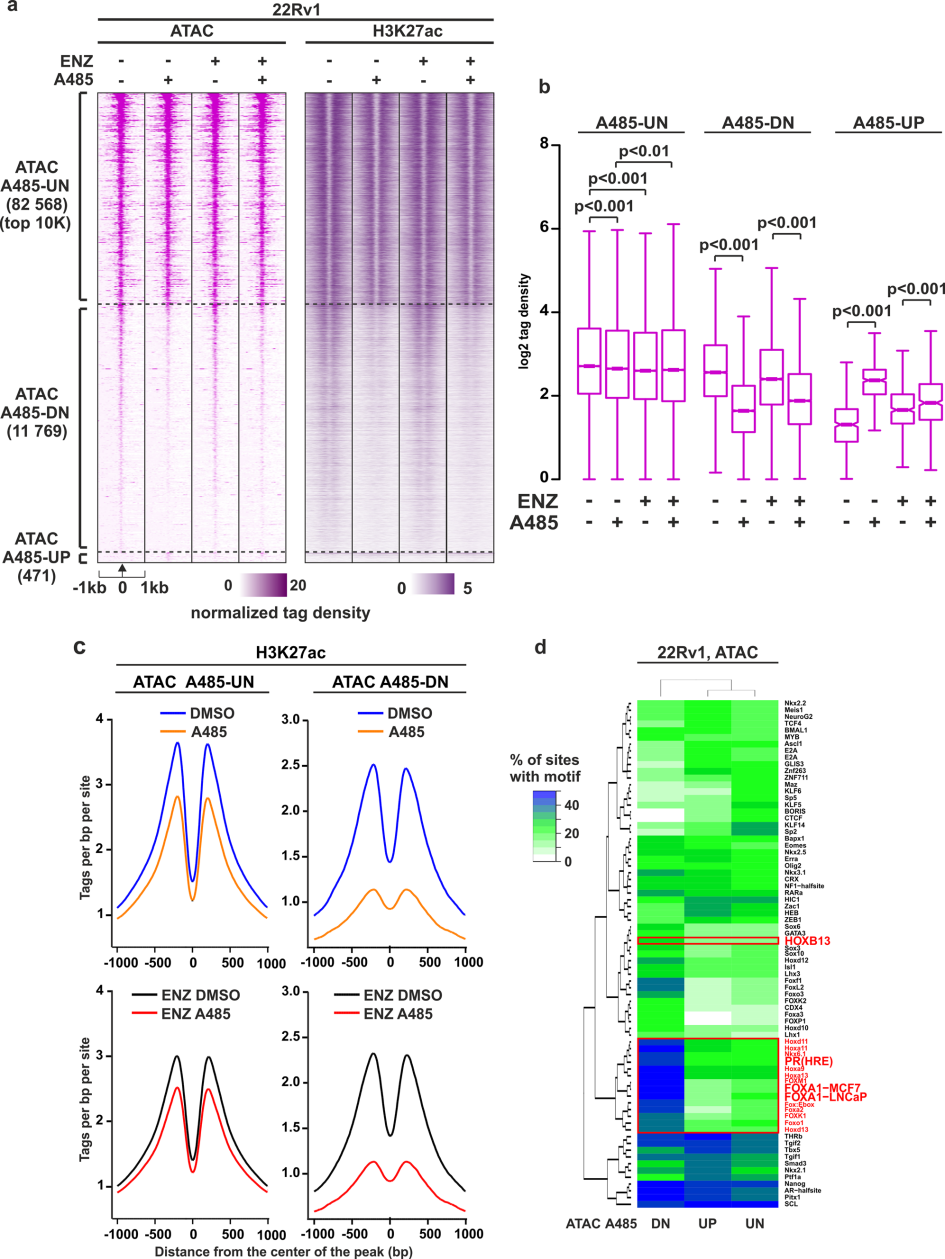
Accessible chromatin sites sensitive to A485 are enriched with FOXA1 motifs. **a** ATAC-seq, and H3K27 ChIP-seq profiles at A485-UN, A485-DN and A485-UP sites in ENZ-naïve and ENZ-exposed 22Rv1 cells. ATAC A485-UN represents unchanged, ATAC A485-UP increased and ATAC A485-DN decreased open chromatin sites. Each heatmap represents ± 1 kb around the center of the ATAC peak. Binding intensity (tags per bp per site) scale is noted below on a linear scale. **b** Box plots represent the normalized log2 tag density of ATAC-seq at indicated sites. Statistical significance calculated using One-way ANOVA with Bonferroni post hoc test. **c** Aggregate plots represent the binding intensity (tags per bp per site) of H3K27ac ChIP-seq at indicated sites in ENZ-naïve (upper) and ENZ-exposed (lower) 22Rv1 cells. Each aggregate plot represents ± 1 kb around the center of the ATAC peak. All heatmaps, box plots, and aggregate plots are normalized to a total of 10 million reads. **d** De novo motif enrichment at indicated open chromatin sites in 22Rv1 cells. Enrichment is displayed as a heatmap representing % of sites with motif. The scale is displayed on the side of the heatmap with white–green–blue indicating the prevalence of enrichment. Red rectangle highlights relevant motifs

As chromatin accessibility plays a crucial role in modulating GR binding in PCa cells [29] (Fig. 3), we sought to investigate the global impact of A485 on chromatin accessibility. Remarkably, A485 treatment led to a clear reduction in chromatin accessibility in both 22Rv1 and VCaP cell lines (Fig. 4a, b, Supplementary Fig. S8d-e). In 22Rv1 cells, just under 12,000 open chromatin sites showed reduced chromatin accessibility upon A485 exposure (ATAC-A485-DN) (Fig. 4a, b). This reduction was accompanied by a simultaneous decrease in the levels of H3K27ac (Fig. 4a and c, Supplementary Fig. S8a-b). The H3K27ac enrichment at ATAC-A485-DN sites was more prominently decreased compared to random set of open chromatin sites (Supplementary Fig. S8c). This was not observed in ATAC-A485-UN sites. Similarly, over 13,000 open chromatin sites showed reduced chromatin accessibility after A485 treatment in VCaP cells (Supplementary Fig. S8d-e). This suggests that, in addition to GR, other TFs depend on EP300/CREBBP acetyltransferase activity to create or maintain an open chromatin configuration. Consequently, we conducted de novo motif analysis to identify these TFs that bind to chromatin sites sensitive to EP300/CREBBP acetyltransferase inhibition. Intriguingly, the open chromatin sites regulated by A485 (ATAC-A485-DN) were found to be enriched with GRE as well as FOXA1 and HOXB13 motifs (Fig. 4d, Supplementary Fig. S8f, Supplementary Table S5). Especially, the FOXA1 motif exhibited a high enrichment at A485-regulated open chromatin sites (Fig. 4d). Furthermore, the FOXA1 motif was also prevalently enriched at A485-DN GRBs in 22Rv1 cells (Fig. 3c). These results suggest that FOXA1 signaling could be sensitive to EP300/CREBBP acetyltransferase inhibition.

### FOXA1 chromatin binding is drastically suppressed upon EP300/CREBBP acetyltransferase inhibition

**Fig. 5:**
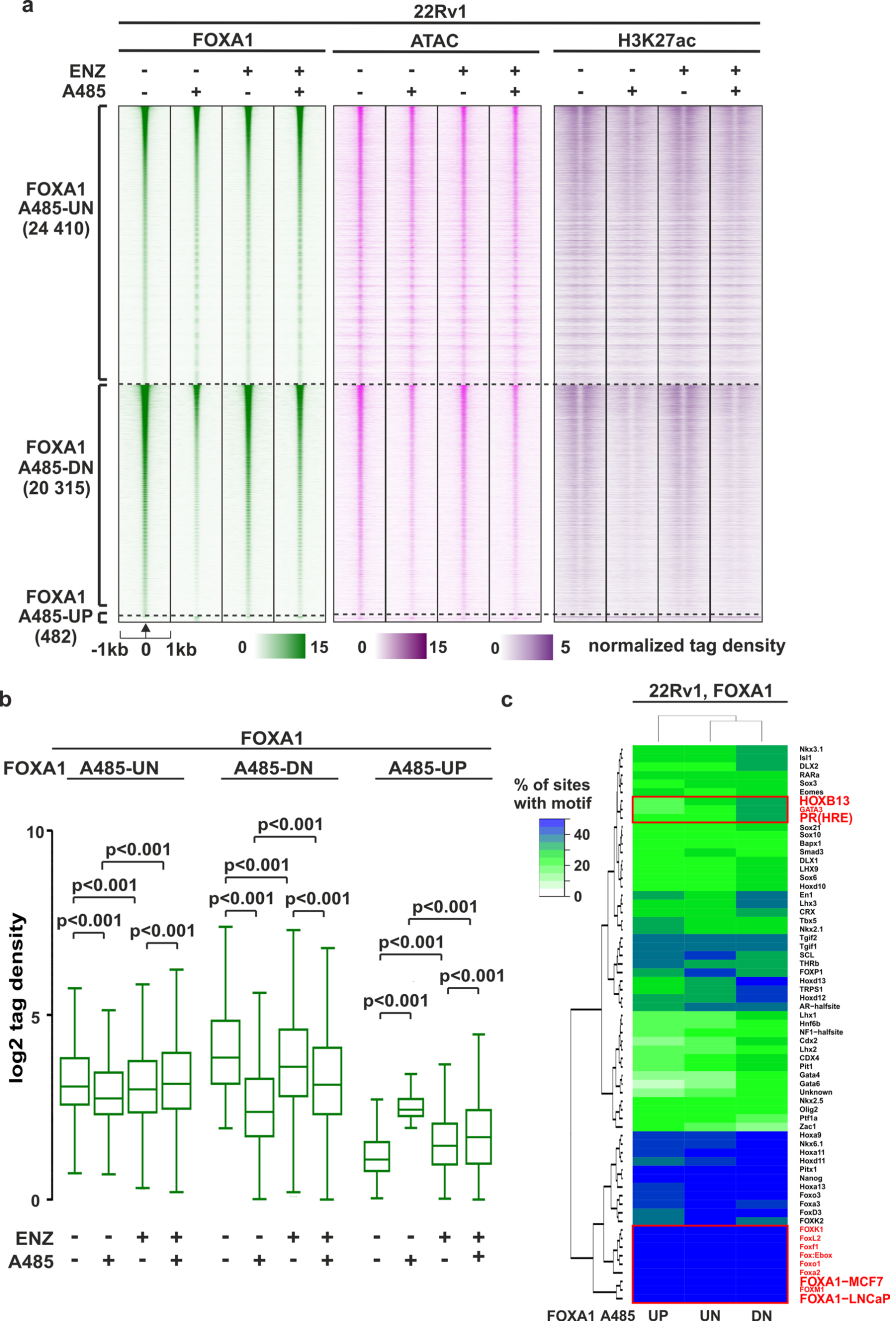
FOXA1 chromatin binding is suppressed upon A485 exposure. **a** FOXA1 ChIP-seq, ATAC-seq, and H3K27 ChIP-seq profiles at A485-UN, A485-DN and A485-UP sites in ENZ-naïve and ENZ-exposed 22Rv1 cells. FOXA1 A485-UN represents unchanged, FOXA1 A485-UP increased and FOXA1 A485-DN decreased FOXA1 binding sites. Each heatmap represents ± 1 kb around the center of the FOXA1 peak. Binding intensity (tags per bp per site) scale is noted below on a linear scale. **b** Box plots represent the normalized log2 tag density of FOXA1 ChIP-seq at indicated sites. Statistical significance calculated using One-way ANOVA with Bonferroni post hoc test. All heatmaps and box plots are normalized to a total of 10 million reads. **c** De novo motif enrichment at indicated FOXA1 sites in 22Rv1 cells. Enrichment is displayed as a heatmap representing % of sites with motif. The scale is displayed on the side of the heatmap with white–green–blue indicating the prevalence of enrichment. Red rectangle highlights relevant motifs

The loss of GR binding in response to A485 treatment suggests that other TFs initially recruit EP300 to the GRBs, thereby facilitating chromatin accessibility. Given the significant enrichment of the FOXA1 motif at GRBs and open chromatin sites regulated by A485 (Figs. 3c, 4d), we conducted FOXA1 ChIP-seq to examine the influence of A485 on FOXA1 chromatin occupancy. We observed a significant decrease in FOXA1 binding; approximately 45% of all FOXA1 binding sites were significantly reduced upon A485 treatment (FOXA1-A485-DN) (Fig. 5a, b). Interestingly, the FOXA1-A485-DN sites showed the highest occupancy of FOXA1 prior to A485 exposure. In line with the GR findings, the FOXA1-A485-DN sites exhibited a reduction in both chromatin accessibility and H3K27ac enrichment (Fig. 5a, Supplementary Fig. S9a-d). Moreover, the open chromatin sites that were downregulated by A485 (ATAC-A485-DN) (Fig. 4a) displayed a substantial occupancy of FOXA1 prior to A485 exposure and a significant reduction of FOXA1 binding after A485 treatment (Supplementary Fig. S9e). Importantly, A485 did not predominantly influence the protein levels of FOXA1 as it did AR protein levels (Supplementary Fig. S7f). Furthermore, de novo motif analysis showed that FOXA1 binding sites were primarily enriched with motifs associated with the FOX-protein family of TFs (Fig. 5c, Supplementary Table S5). In addition, FOXA1-A485-DN showed enrichment of GRE sequences. Interestingly, the intricate relationship between EP300 and FOXA1 is evident in PCa patients, where a strong positive correlation is observed between the expression levels of *FOXA1* and *EP300/CREBBP* (Supplementary Fig. S9f), particularly in primary and AR-positive CRPC patients. Moreover, like *FOXA1-EP300/CREBBP*, the expression levels of *FOXA1* and *AR* displayed a strong positive correlation (Supplementary Fig. S9g), particularly in primary and AR-positive CRPC patients. Intriguingly, FOXA1 binding is severely decreased at the enhancer region regulating *AR*-gene expression [53, 54] upon A485 treatment (Supplementary Fig. S9h). This suggests that the loss of FOXA1 binding contributes to the decreased *AR*-gene regulation and the subsequent inhibition of AR signaling.

### GR binding sites exhibit prevalent loss of FOXA1 chromatin occupancy upon A485 exposure

**Fig. 6:**
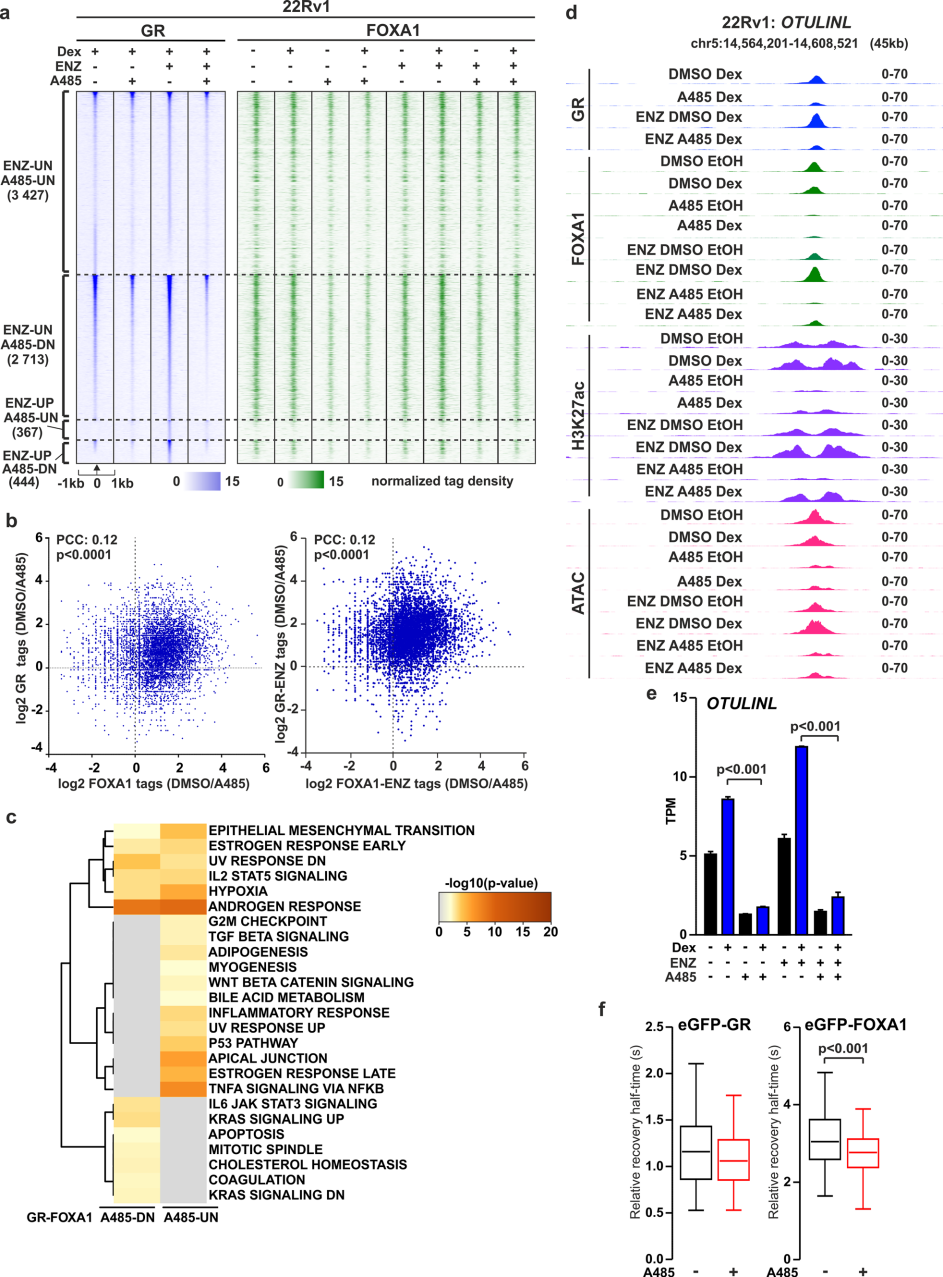
A485-mediated loss of GR and FOXA1 chromatin binding occurs concordantly. **a** GR ChIP-seq and FOXA1 ChIP-seq profiles at ENZ-UN A485-UN, ENZ-UN A485-DN, ENZ-UP A485-UN and ENZ-UP A485-DN GR binding sites in ENZ-naïve and ENZ-exposed 22Rv1 cells. UN represents unchanged, UP increased and DN decreased GRBs. Each heatmap represents ± 1 kb around the center of the GR peak. Binding intensity (tags per bp per site) scale is noted below on a linear scale. **b** Scatter plots of GR (Y-axis) and FOXA1 (X-axis) ChIP-seq log2 tag density for DMSO/A485 in ENZ-naïve (left) and ENZ-exposed (right) 22Rv1 cells. **c** Hallmark Gene Sets pathway analysis of genes associated with A485-UN and A485-DN GR-FOXA1 sites (log2FC > 1, n = 1400) in 22Rv1 cells. **d** Genome browser track examples of GR, FOXA1 and H3K27ac ChIP-seq, and ATAC-seq at *OTULINL* locus in 22Rv1 cells. **e** Bar graph depicts *OTULINL* expression levels in 22Rv1 cells from RNA-seq data. Data shown as transcripts per million (TPM). Statistical significance calculated with One-way ANOVA with Bonferroni post hoc test. n = 2. **f** Box plots represent the relative half-recovery times of eGFP-GR and -FOXA1 in DMSO (black) and A485 (red) conditions. Statistical significance was calculated using an unpaired t-test

FOXA1 is known to regulate GR action in PCa cells and is prevalently bound to GRBs [29]. Intriguingly, ChIP-seq analyses indicated a significant reduction in FOXA1 binding upon A485 treatment at GRBs, especially at A485-DN GRBs (Fig. 6a, Supplementary Fig. S10a). This suggests that the restriction of GR binding to chromatin after A485 treatment is linked to the reduction of FOXA1 binding. Our previous findings have indicated that loss of FOXA1 potentiates GR transcriptional activity due to FOXA1-mediated repression of *NR3C1* through corepressor TLE3 [29]. This indicates that EP300/CREBBP acetyltransferase inhibition results in the loss of FOXA1 chromatin opening capabilities while retaining its repressive potential. Indeed, differing from *AR*-gene regulating enhancer, FOXA1 binding is retained after A485 treatment at FOXA1-bound enhancers related to *NR3C1*-repression through TLE3 (Supplementary Fig. S10b) [29]. Interestingly, a cross-comparison of the fold change (DMSO-vs-A485) between FOXA1 and GR at GRBs showed numerous sites where the loss occurs concurrently (Fig. 6b). To gain further insights into the functional implications of the concurrently reduced chromatin sites, we focused on the GRBs that showed a clear A485-reduced change for both FOXA1 and GR (see Materials and Methods for details). Pathway analyses revealed that the genes associated with these GRBs (GR-FOXA1 A485-DN) were involved in pathways related to apoptosis, mitosis and KRAS signaling (Fig. 6c, Supplementary Table S2). As an example, upon A485 exposure, *OTULINL* locus displayed inhibition in GR and FOXA1 chromatin binding, reduction in H3K27ac enrichment and chromatin accessibility, as well as repression of Dex-induction (Fig. 6d, e). To evaluate the clinical relevance, we created GR-FOXA1 signature (see Materials and Methods for details). TCGA PCa patients with low GR-FOXA1 signature showed significantly improved disease-free survival compared patients with high GR-FOXA1 signature (Supplementary Fig. S10c). For SU2C PCa patients, there were no significant difference between the low and high GR-FOXA1 signature, although the trend was like in TCGA dataset. This indicates, at least partial clinical relevance of GR targets modulated by the EP300/CREBBP acetyltransferase inhibition through the loss of FOXA1 binding.

Since the chromatin binding of both GR and FOXA1 was substantially restricted after A485 treatment we sought to complement this notion in live cells by performing FRAP experiments. For this, we transfected 22Rv1 cells with eGFP-tagged GR or FOXA1, as well as with TATA-box binding-protein (TBP) as a control (Supplementary Fig. S11a). We observed notable differences in the recovery processes among the proteins. TBP showed the smallest mobile fraction and recovery rate compared to GR and FOXA1 (Supplementary Fig. S11b). FRAP assays showed that the half-recovery times of only FOXA1 significantly decreased in the presence of A485 (Fig. 6f, Supplementary Fig. S11e). There was no significant change with GR or TBP (Fig. 6f, Supplementary Fig. S11c-d). Our live cell data thus support FOXA1 ChIP-seq data showing decreased association of FOXA1 with chromatin upon EP300/CREBBP acetyltransferase inhibition. Finally, since GR is not influenced by A485 in live cells, it could be postulated that decrease in GR chromatin binding is derived from the alterations in the action of FOXA1.

## Discussion

It has been suggested that in cancer therapy, it would be more efficient to inhibit the action of coregulators rather than individual TFs [55]. The rationale behind this lies in the fact that many cancer-related TFs, such as steroid receptors, MYC, and NF-κB, all utilize the same set of coregulators. Furthermore, coactivators have been shown to be 100-fold less abundant than corepressors [56]. These investigations highlight that inhibiting the activity of coactivators would efficiently restrict the action of multiple oncogenic TFs. Although several small-molecule drugs against corepressors have already been developed for clinical use [15], modulators against coactivators are not far behind. The most advanced progress has been made with inhibitors targeting bromodomain and extra-terminal (BET) domain family members in inflammation and cancers [57–59]. In PCa, BET inhibitors are effective in inhibiting AR signaling. Moreover, EP300/CREBBP has recently been targeted by small-molecule inhibitors [16, 17]. These include both acetyltransferase and BRD inhibitors, A485, and CCS1477, respectively, with the latter being evaluated in clinical trials in patients with solid tumors, including CRPC tumors (NCT03568656). While the influence of BET inhibitors on AR chromatin binding has been investigated in PCa [57, 58], the effect of EP300/CREBBP inhibitors has remained elusive. To fill this knowledge gap, we characterized the impact of A485 on steroid receptor signaling in 22Rv1 and VCaP cells.

We chose to utilize the EP300/CREBBP acetyltransferase inhibitor A485 in our study for several reasons: firstly, the treatment of A485 resembles that of EP300/CREBBP knockout [24], secondly, it impairs the expression of oncogenic TFs [52], and finally, among the tested inhibitors, it showed the most prominent effect on 22Rv1 cell proliferation. In terms of AR signaling, we found that EP300/CREBBP acetyltransferase inhibition drastically decreased both the binding of AR and the DHT-regulated transcriptome. Interestingly, this effect was more prominent in VCaP cells compared to 22Rv1 cells. One potential reason for this difference could be the amplification of the *AR*-gene in VCaP cells, which may contribute to the observed phenomenon.

**Fig. 7:**
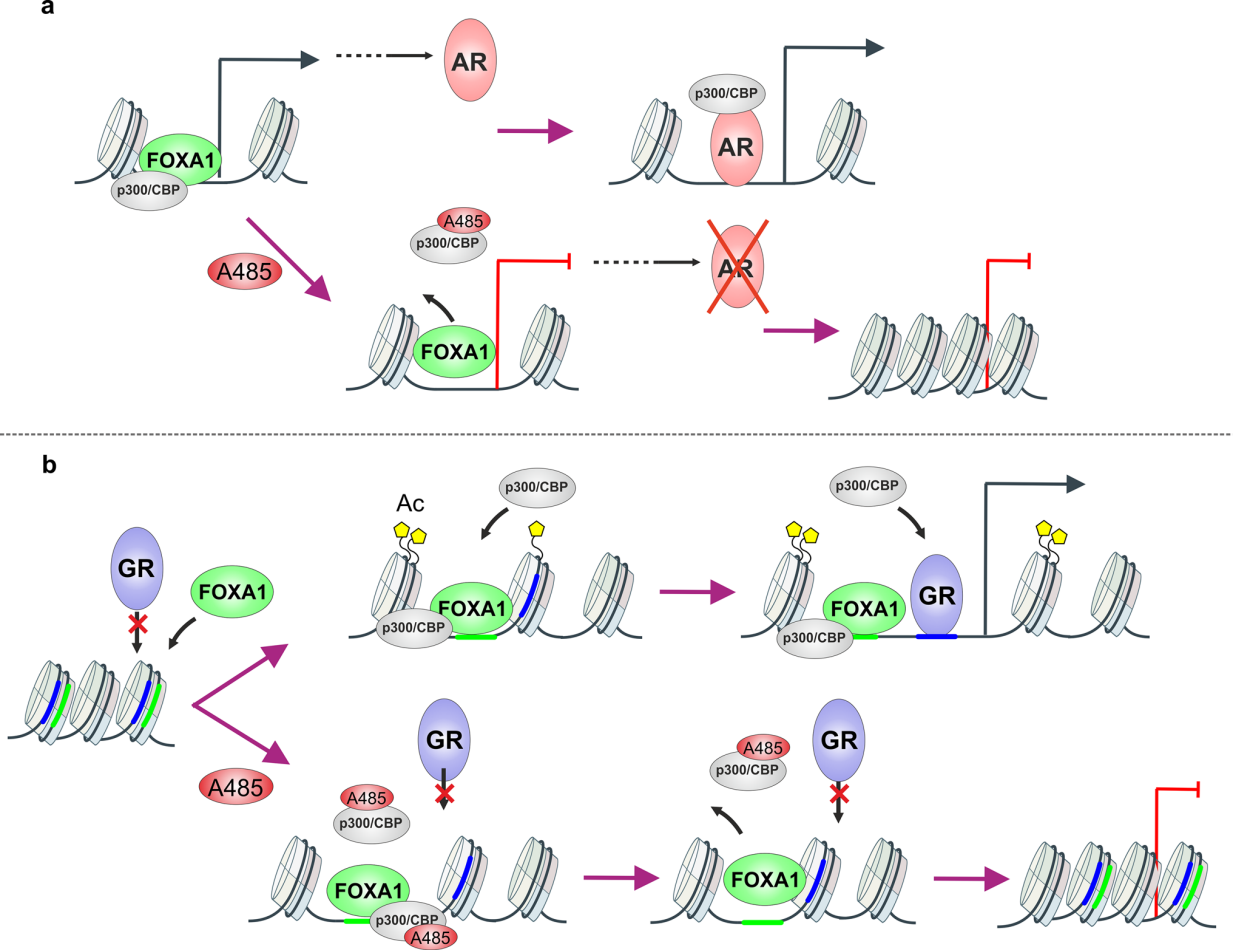
Model of A485 mediated repression of AR and GR transcriptional activity. **a** A485 restricts AR chromatin occupancy and transcriptional activity by suppressing the *AR*-gene. **b** A485 restricts GR chromatin occupancy and transcriptional activity through the loss of FOXA1 chromatin occupancy

Based on our data, the mechanism of *AR*-gene repression occurs, at least partially, through the A485-mediated reduction of FOXA1 binding at the *AR*-gene enhancer (Fig. 7a). This enhancer is located more than 600 kb away from the AR locus, yet it loops to the gene promoter [54]. Furthermore, this enhancer is frequently amplified in CRPC patients, often coinciding with *AR*-gene amplification [53]. While the depletion of FOXA1 alone through siRNA does not lead to a decrease in AR protein levels [60], it is likely that other TFs also contribute to the regulation of the *AR*-gene and its repression after exposure to A485 [54]. Nevertheless, the inhibition of EP300/CREBBP activity would presumably offer a particularly effective therapeutic approach for CRPC patients with AR enhancer amplification. Notably, A485 exposure has also demonstrated the ability to downregulate estrogen receptor protein levels in breast cancer [61], and EP300/CREBBP acetyltransferase inhibition curtails estrogen signaling through FOXA1-bound enhancers [62]. This underscores the broader potential of EP300/CREBBP inhibition in steroid receptor-driven cancers, with FOXA1 signaling occupying a central role in these effects.

Interestingly, the impact of A485 on GR signaling presents a distinct contrast to its effect on AR. Rather than influencing GR transcript or protein levels, A485 treatment leads to a significant reduction in chromatin accessibility at enhancers where GR binds, ultimately inhibiting receptor binding (Fig. 7b). This decline in chromatin accessibility can be attributed to the diminished occupancy of FOXA1 at these enhancers. Consequently, A485 exposure triggers a decrease in the occurrence of H3K27ac and chromatin accessibility, which in turn leads to the loss of FOXA1 binding. It can be postulated that the decreased FOXA1 occupancy and chromatin accessibility results in the inability of GR to occupy these sites, considering that GR primarily binds to open chromatin sites in PCa cells [29]. Thus, the loss of both FOXA1 binding and EP300/CREBBP activity curtails the signaling of both AR and GR. However, the underlying mechanism of action differs significantly between the two receptors (Fig. 7a, b).

FOXA1, a recognized pioneer factor, maintains a complex relationship with AR in PCa, wherein it can both pioneer and mask the binding of the receptor to chromatin [60]. Furthermore, activated AR has the capacity to pioneer FOXA1 binding [63]. We have previously demonstrated that the depletion of FOXA1 enhances GR activity in PCa cells by virtue of FOXA1-mediated inhibition of *NR3C1* expression through the corepressor TLE3 [29]. However, a question arises: why does A485-mediated loss of FOXA1 signaling not lead to the potentiation of GR binding? While EP300/CREBBP acetyltransferase inhibition affects nearly half of FOXA1-bound sites, the binding of FOXA1 remains intact at enhancer responsible for the repression of *NR3C1*. Consequently, A485 treatment does not impede the repressive role of FOXA1. Interestingly, it has been demonstrated that EP300/CREBBP can engage in transcriptional repression alongside Polycomb-complex [64], and this repression occurs independently of EP300/CREBBP’s acetyltransferase activity.

In addition to its influence on histone acetylation and subsequent chromatin accessibility, A485 treatment also leads to a reduction in the acetylation levels of various TFs and coregulators [24]. Both AR and GR undergo acetylation at their hinge domains; however, this modification yields divergent effects on receptor function [65]. While acetylation enhances the transcriptional activity of AR, it diminishes the functional potency of GR. This implies that a portion of A485’s repressive impact on AR signaling may stem from the absence of receptor acetylation. Given that the absence of GR’s acetylation enhances its transcriptional activity, it is likely that A485-induced receptor deacetylation has minimal influence on the observed effects with GR. Like GR, FOXA1’s activity is attenuated by acetylation [66]. Acetylation sites in FOXA1 reside within its DNA-binding domain, and this modification is hindered by FOXA1’s engagement with chromatin. Considering FOXA1’s pre-existing binding to GRBs prior to A485 exposure, the impact of A485 on FOXA1 signaling remains independent of its acetylation status.

While the impact of A485 on GR and AR signaling, as well as chromatin accessibility, is similar in both 22Rv1 and VCaP cells, certain differences exist. Besides A485-mediated differences in AR chromatin binding, the A485 exposure more profoundly represses the GR-regulated transcriptome in VCaP compared to 22Rv1 cells. This implies that the consequences of EP300/CREBBP acetyltransferase inhibition by A485 on H3K27ac enrichment and FOXA1 chromatin binding, not profiled in VCaP cells, may vary between the cell lines. However, the diminishing effect of A485 on H3K27ac protein levels is comparable between the two cell lines. Furthermore, given the similarity in FOXA1-mediated repression of *NR3C1* expression between VCaP and 22Rv1 cells [29], it is plausible that the GR-FOXA1 relationship in A485-exposed VCaP cells mirrors that observed in 22Rv1 cells. Nevertheless, the predominant impact of A485 on FOXA1 chromatin binding warrants further validation in other prostate cancer cell lines in future studies.

The lack of effective therapies has been a key factor contributing to the low survival rates observed in CRPC and its therapy-resistant variants [2, 4]. Intriguingly, published studies and our data indicate that inhibiting EP300/CREBBP activity holds promise as a therapeutic option. These compounds can effectively target multiple signaling pathways that play a role in therapy resistance, including those involving AR-V7 and GR [17, 29]. However, a notable question that remains is the extent of EP300/CREBBP’s involvement in AR-negative PCa. This question arises from observations that CCS1477 has a limited impact on the proliferation of AR-negative prostate cancer cells [17]. Given that AR-negative prostate cancer accounts for approximately 50% of CRPC cases and represents one of the most lethal forms of the disease [67], further research is imperative to elucidate the role of EP300/CREBBP in this context.

### Supplementary Information

Below is the link to the electronic supplementary material.

Supplementary file1 (PDF 2820 KB)

Supplementary file2 (XLSX 40883 KB)

Supplementary file3 (XLSX 34 KB)

Supplementary file4 (XLSX 12 KB)

Supplementary file5 (XLSX 66130 KB)

Supplementary file6 (XLSX 402 KB)

## Data Availability

The generated ChIP-seq, RNA-seq and ATAC-seq datasets have been submitted to the NCBI Gene Expression Omnibus database (http://www.ncbi.nlm.nih.gov/geo/). Accession code: GSE245969.

## References

[1] Paakinaho V, Palvimo JJ (2021). Genome-wide crosstalk between steroid receptors in breast and prostate cancers. Endocr Relat Cancer.

[2] Swami U, McFarland TR, Nussenzveig R, Agarwal N (2020). Advanced prostate cancer: treatment advances and future directions. Trends Cancer.

[3] Abida W, Cyrta J, Heller G, Prandi D, Armenia J, Coleman I (2019). Genomic correlates of clinical outcome in advanced prostate cancer. Proc Natl Acad Sci U S A.

[4] Carceles-Cordon M, Kelly WK, Gomella L, Knudsen KE, Rodriguez-Bravo V, Domingo-Domenech J (2020). Cellular rewiring in lethal prostate cancer: the architect of drug resistance. Nat Rev Urol.

[5] Joseph JD, Lu N, Qian J, Sensintaffar J, Shao G, Brigham D (2013). A clinically relevant androgen receptor mutation confers resistance to second-generation antiandrogens enzalutamide and ARN-509. Cancer Discov.

[6] Antonarakis ES, Lu C, Wang H, Luber B, Nakazawa M, Roeser JC (2014). AR-V7 and resistance to enzalutamide and abiraterone in prostate cancer. N Engl J Med.

[7] Arora VK, Schenkein E, Murali R, Subudhi SK, Wongvipat J, Balbas MD (2013). XGlucocorticoid receptor confers resistance to antiandrogens by bypassing androgen receptor blockade. Cell..

[8] Kalfeist L, Galland L, Ledys F, Ghiringhelli F, Limagne E, Ladoire S (2022). Impact of glucocorticoid use in oncology in the immunotherapy era. Cells..

[9] Serritella AV, Shevrin D, Heath EI, Wade JL, Martinez E, Anderson A (2022). Phase I/II trial of enzalutamide and mifepristone, a glucocorticoid receptor antagonist, for metastatic castration-resistant prostate cancer. Clin Cancer Res.

[10] Jiang J, Yuan J, Hu Z, Xu M, Zhang Y, Long M (2022). Systematic pan-cancer characterization of nuclear receptors identifies potential cancer biomarkers and therapeutic targets. Cancer Res.

[11] Burris TP, de Vera IMS, Cote I, Flaveny CA, Wanninayake US, Chatterjee A (2023). International union of basic and clinical pharmacology cxiii: nuclear receptor superfamily-update 2023. Pharmacol Rev.

[12] Lempiäinen JK, Niskanen EA, Vuoti KM, Lampinen RE, Göös H, Varjosalo M (2017). Agonist-specific protein interactomes of glucocorticoid and androgen receptor as revealed by proximity mapping. Mol Cell Proteomics.

[13] Valencia AM, Kadoch C (2019). Chromatin regulatory mechanisms and therapeutic opportunities in cancer. Nat Cell Biol.

[14] Jafari H, Hussain S, Campbell MJ (2022). Nuclear receptor coregulators in hormone-dependent cancers. Cancers (Basel)..

[15] Bates SE (2020). Epigenetic therapies for cancer. N Engl J Med.

[16] Lasko LM, Jakob CG, Edalji RP, Qiu W, Montgomery D, Digiammarino EL (2017). Discovery of a selective catalytic p300/CBP inhibitor that targets lineage-specific tumours. Nature.

[17] Welti J, Sharp A, Brooks N, Yuan W, McNair C, Chand SN (2021). Targeting the p300/cbp axis in lethal prostate cancer. Cancer Discov.

[18] Ogryzko VV, Schiltz RL, Russanova V, Howard BH, Nakatani Y (1996). The transcriptional coactivators p300 and CBP are histone acetyltransferases. Cell.

[19] Vo N, Goodman RH (2001). CREB-binding protein and p300 in transcriptional regulation. J Biol Chem.

[20] Comuzzi B, Nemes C, Schmidt S, Jasarevic Z, Lodde M, Pycha A (2004). The androgen receptor co-activator CBP is up-regulated following androgen withdrawal and is highly expressed in advanced prostate cancer. J Pathol.

[21] Debes JD, Sebo TJ, Lohse CM, Murphy LM, Haugen DAL, Tindall DJ (2003). p300 in prostate cancer proliferation and progression. Cancer Res.

[22] Millán-Zambrano G, Burton A, Bannister AJ, Schneider R (2022). Histone post-translational modifications - cause and consequence of genome function. Nat Rev Genet.

[23] Zucconi BE, Makofske JL, Meyers DJ, Hwang Y, Wu M, Kuroda MI (2019). Combination targeting of the bromodomain and acetyltransferase active site of p300/CBP. Biochemistry.

[24] Weinert BT, Narita T, Satpathy S, Srinivasan B, Hansen BK, Schölz C (2018). Time-resolved analysis reveals rapid dynamics and broad scope of the CBP/p300 acetylome. Cell.

[25] Kikuchi M, Morita S, Wakamori M, Sato S, Uchikubo-Kamo T, Suzuki T (2023). Epigenetic mechanisms to propagate histone acetylation by p300/CBP. Nat Commun.

[26] Wimalasena VK, Wang T, Sigua LH, Durbin AD, Qi J (2020). Using chemical epigenetics to target cancer. Mol Cell.

[27] Yu X, Yi P, Hamilton RA, Shen H, Chen M, Foulds CE (2020). Structural insights of transcriptionally active, full-length androgen receptor coactivator complexes. Mol Cell.

[28] Furlan T, Kirchmair A, Sampson N, Puhr M, Gruber M, Trajanoski Z (2021). MYC-mediated ribosomal gene expression sensitizes enzalutamide-resistant prostate cancer cellS TO EP300/CREBBP inhibitors. Am J Pathol.

[29] Helminen L, Huttunen J, Tulonen M, Aaltonen N, Niskanen EA, Palvimo JJ (2024). Chromatin accessibility and pioneer factor FOXA1 restrict glucocorticoid receptor action in prostate cancer. Nucleic Acids Res..

[30] McDowell IC, Barrera A, D’Ippolito AM, Vockley CM, Hong LK, Leichter SM (2018). Glucocorticoid receptor recruits to enhancers and drives activation by motif-directed binding. Genome Res.

[31] Karvonen U, Kallio PJ, Jänne OA, Palvimo JJ (1997). Interaction of androgen receptors with androgen response element in intact cells. Roles of amino- and carboxyl-terminal regions and the ligand. J Biol Chem..

[32] Launonen KM, Paakinaho V, Sigismondo G, Malinen M, Sironen R, Hartikainen JM (2021). Chromatin-directed proteomics-identified network of endogenous androgen receptor in prostate cancer cells. Oncogene.

[33] Dobin A, Davis CA, Schlesinger F, Drenkow J, Zaleski C, Jha S (2013). STAR: ultrafast universal RNA-seq aligner. Bioinformatics.

[34] Love MI, Huber W, Anders S (2014). Moderated estimation of fold change and dispersion for RNA-seq data with DESeq2. Genome Biol.

[35] Heinz S, Benner C, Spann N, Bertolino E, Lin YC, Laslo P (2010). Simple combinations of lineage-determining transcription factors prime cis-regulatory elements required for macrophage and B cell identities. Mol Cell.

[36] Zhou Y, Zhou B, Pache L, Chang M, Khodabakhshi AH, Tanaseichuk O (2019). Metascape provides a biologist-oriented resource for the analysis of systems-level datasets. Nat Commun.

[37] Cerami E, Gao J, Dogrusoz U, Gross BE, Sumer SO, Aksoy BA (2012). The cBio cancer genomics portal: an open platform for exploring multidimensional cancer genomics data. Cancer Discov.

[38] Li R, Zhu J, Zhong WD, Jia Z. PCaDB - a comprehensive and interactive database for transcriptomes from prostate cancer population cohorts. bioRxiv [Internet]. 2021 Jan 1;2021.06.29.449134. Available from: http://biorxiv.org/content/early/2021/08/21/2021.06.29.449134.abstract

[39] Bolis M, Bossi D, Vallerga A, Ceserani V, Cavalli M, Impellizzieri D (2021). Dynamic prostate cancer transcriptome analysis delineates the trajectory to disease progression. Nat Commun.

[40] Nusinow DP, Szpyt J, Ghandi M, Rose CM, McDonald ER, Kalocsay M (2020). Quantitative proteomics of the cancer cell line encyclopedia. Cell.

[41] Paakinaho V, Kaikkonen S, Makkonen H, Benes V, Palvimo JJ (2014). SUMOylation regulates the chromatin occupancy and anti-proliferative gene programs of glucocorticoid receptor. Nucleic Acids Res.

[42] Buenrostro JD, Wu B, Chang HY, Greenleaf WJ (2015). ATAC-seq: a method for assaying chromatin accessibility genome-wide. Curr Protoc Mol Biol..

[43] Paakinaho V, Lempiäinen JK, Sigismondo G, Niskanen EA, Malinen M, Jääskeläinen T (2021). SUMOylation regulates the protein network and chromatin accessibility at glucocorticoid receptor-binding sites. Nucleic Acids Res.

[44] Langmead B, Trapnell C, Pop M, Salzberg SL (2009). Ultrafast and memory-efficient alignment of short DNA sequences to the human genome. Genome Biol.

[45] Langmead B, Salzberg SL (2012). Fast gapped-read alignment with Bowtie 2. Nat Methods.

[46] Parolia A, Cieslik M, Chu SC, Xiao L, Ouchi T, Zhang Y (2019). Distinct structural classes of activating FOXA1 alterations in advanced prostate cancer. Nature.

[47] Kron KJ, Murison A, Zhou S, Huang V, Yamaguchi TN, Shiah YJ (2017). TMPRSS2-ERG fusion co-opts master transcription factors and activates NOTCH signaling in primary prostate cancer. Nat Genet.

[48] Long Q, Xu J, Osunkoya AO, Sannigrahi S, Johnson BA, Zhou W (2014). Global transcriptome analysis of formalin-fixed prostate cancer specimens identifies biomarkers of disease recurrence. Cancer Res.

[49] Cancer Genome Atlas Research Network (2015). The molecular taxonomy of primary prostate cancer. Cell.

[50] Ferrie JJ, Karr JP, Graham TGW, Dailey GM, Zhang G, Tjian R (2024). p300 is an obligate integrator of combinatorial transcription factor inputs. Mol Cell.

[51] Narita T, Ito S, Higashijima Y, Chu WK, Neumann K, Walter J (2021). Enhancers are activated by p300/CBP activity-dependent PIC assembly, RNAPII recruitment, and pause release. Mol Cell.

[52] Hogg SJ, Motorna O, Cluse LA, Johanson TM, Coughlan HD, Raviram R (2021). Targeting histone acetylation dynamics and oncogenic transcription by catalytic P300/CBP inhibition. Mol Cell.

[53] Viswanathan SR, Ha G, Hoff AM, Wala JA, Carrot-Zhang J, Whelan CW (2018). Structural alterations driving castration-resistant prostate cancer revealed by linked-read genome sequencing. Cell.

[54] Takeda DY, Spisák S, Seo JH, Bell C, O’Connor E, Korthauer K (2018). A somatically acquired enhancer of the androgen receptor is a noncoding driver in advanced prostate cancer. Cell.

[55] Lonard DM, O’Malley BW (2016). Molecular pathways: targeting steroid receptor coactivators in cancer. Clin Cancer Res.

[56] Gillespie MA, Palii CG, Sanchez-Taltavull D, Shannon P, Longabaugh WJR, Downes DJ (2020). Absolute quantification of transcription factors reveals principles of gene regulation in erythropoiesis. Mol Cell.

[57] Asangani IA, Dommeti VL, Wang X, Malik R, Cieslik M, Yang R (2014). Therapeutic targeting of BET bromodomain proteins in castration-resistant prostate cancer. Nature.

[58] Faivre EJ, McDaniel KF, Albert DH, Mantena SR, Plotnik JP, Wilcox D (2020). Selective inhibition of the BD2 bromodomain of BET proteins in prostate cancer. Nature.

[59] Gilan O, Rioja I, Knezevic K, Bell MJ, Yeung MM, Harker NR (2020). Selective targeting of BD1 and BD2 of the BET proteins in cancer and immunoinflammation. Science.

[60] Sahu B, Laakso M, Ovaska K, Mirtti T, Lundin J, Rannikko A (2011). Dual role of FoxA1 in androgen receptor binding to chromatin, androgen signalling and prostate cancer. EMBO J.

[61] Waddell A, Mahmud I, Ding H, Huo Z, Liao D (2021). Pharmacological inhibition of CBP/p300 blocks estrogen receptor alpha (ERα) function through suppressing enhancer H3K27 acetylation in luminal breast cancer. Cancers (Basel)..

[62] Bommi-Reddy A, Park-Chouinard S, Mayhew DN, Terzo E, Hingway A, Steinbaugh MJ (2022). CREBBP/EP300 acetyltransferase inhibition disrupts FOXA1-bound enhancers to inhibit the proliferation of ER+ breast cancer cells. PLoS One..

[63] Paakinaho V, Swinstead EE, Presman DM, Grøntved L, Hager GL (2019). Meta-analysis of chromatin programming by steroid receptors. Cell Rep.

[64] Hunt G, Boija A, Mannervik M (2022). p300/CBP sustains Polycomb silencing by non-enzymatic functions. Mol Cell.

[65] Ashton AW, Dhanjal HK, Rossner B, Mahmood H, Patel VI, Nadim M (2022). Acetylation of nuclear receptors in health and disease: an update. FEBS J..

[66] Kohler S, Cirillo LA (2010). Stable chromatin binding prevents FoxA acetylation, preserving FoxA chromatin remodeling. J Biol Chem.

[67] Tang F, Xu D, Wang S, Wong CK, Martinez-Fundichely A, Lee CJ (2022). Chromatin profiles classify castration-resistant prostate cancers suggesting therapeutic targets. Science..

