# Supplementary material for: EP300/CREBBP acetyltransferase inhibition limits steroid receptor and FOXA1 signaling in prostate cancer cells
Source: Cell Mol Life Sci. 2024 Apr 2;81(1):160. doi: 10.1007/s00018-024-05209-z (PMC10987371; doi:10.1007/s00018-024-05209-z)
Supplement: Supplementary file 1 — Supplementary file1 (PDF 2820 KB) [file 18_2024_5209_MOESM1_ESM.pdf]

Supplementary materials for

EP300/CREBBP acetyltransferase inhibition limits steroid receptor and FOXA1 signaling in prostate cancer cells.

Jasmin Huttunen, Niina Aaltonen, Laura Helminen, Kirsi Rilla, Ville Paakinaho

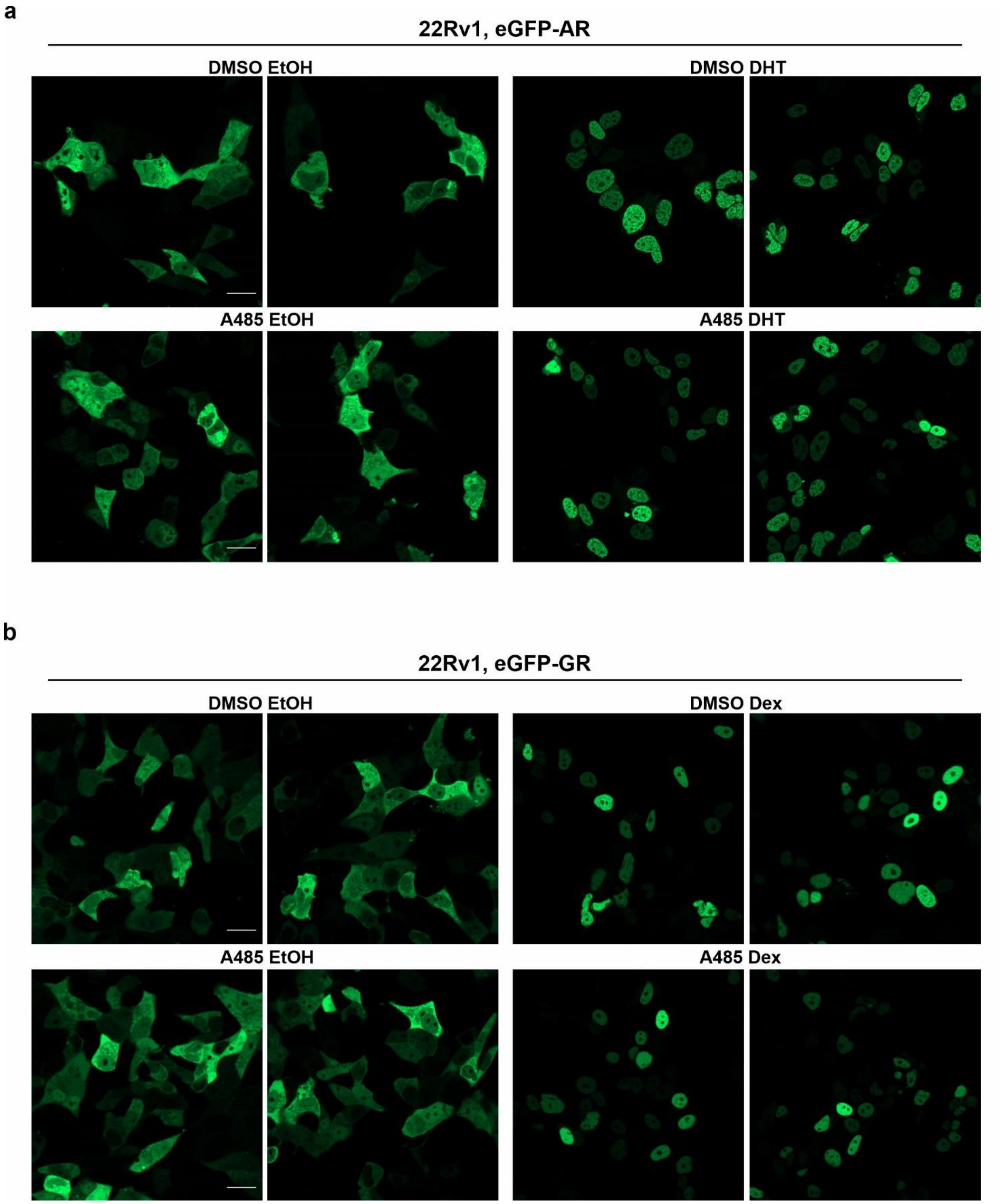

Supplementary Fig. S1. AR and GR are predominantly cytoplasmic without cognate steroid hormone treatment irrespective of A485 treatment. Confocal images of **(a)** *AR* and **(b)** *GR* from 22Rv1 cells. The cells were transfected with eGFP-AR or -GR and treated with DMSO or A485 and vehicle (EtOH) or cognate steroid hormone (Dex or DHT). Scale bar, 20 µm.

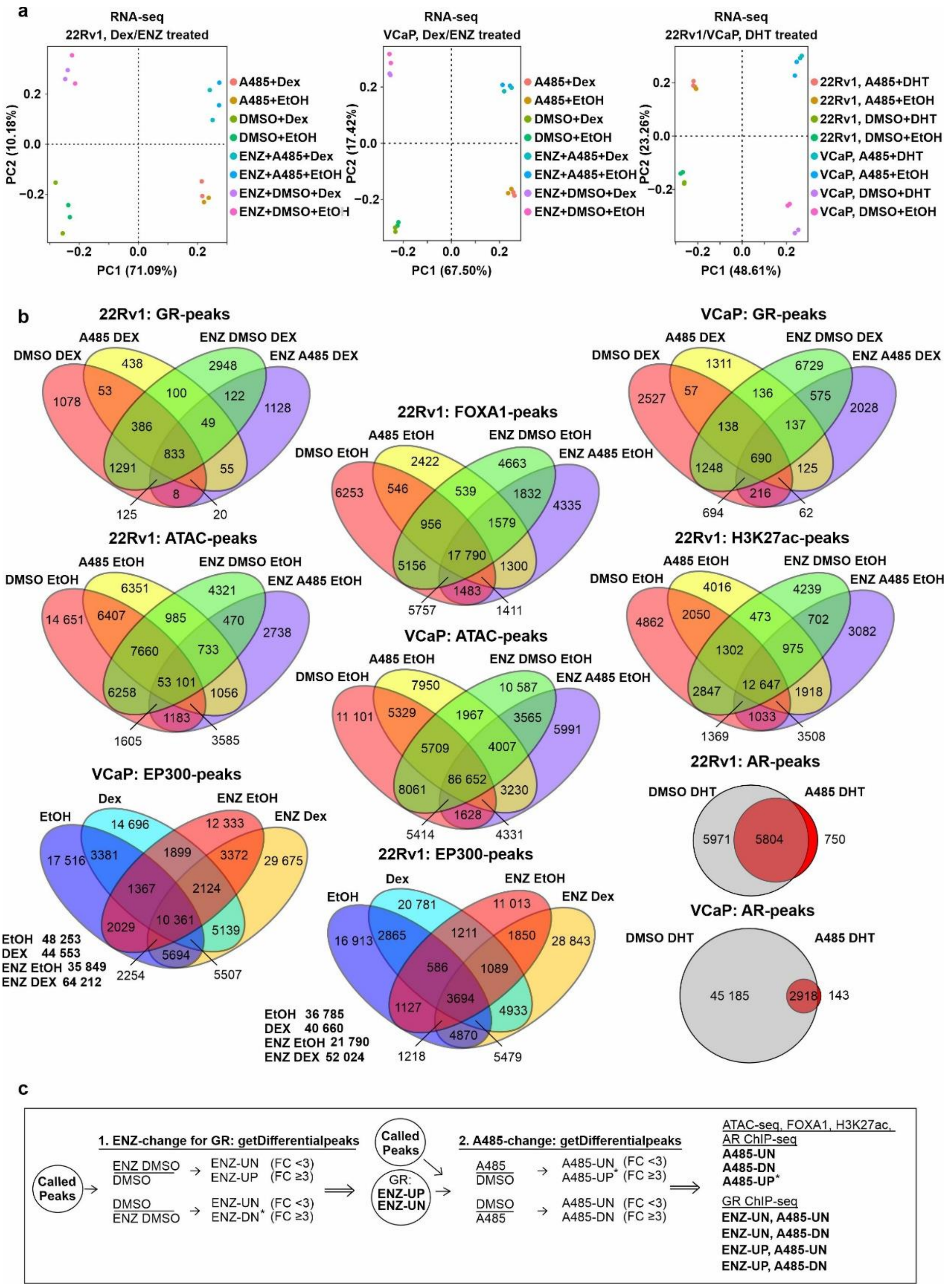

Supplementary Fig. S2. Clustering of expressed genes and overlap of ChIP-seq and ATAC-seq peaks in 22Rv1 and VCaP cells. (a) Principal Component Analysis (PCA) plots depict RNA-seq data derived from 22Rv1 and VCaP cells. PCA is based on the expressed (TPM>0.5) protein-coding genes. Each plot represents two biological replicates per treatment with each treatment color coded. **(b)** Venn diagrams depict the overlap of called peaks for each treatment condition in ATAC-seq and ChIP-seq experiments prior clustering the peaks. **(c)** A graphical representation of ChIP-seq and ATAC-seq peak clustering. For GR ChIP-seq (in 22Rv1 cells), clustering occurs in two steps. In the first step (Part 1), called peaks are clustered based on the effect of ENZ, resulting in two groups: increased (ENZ-UP) and unchanged (ENZ-UN) peaks. In the second step (Part 2), these clusters are further categorized based on the effect of A485 into decreased (A485-DN) and unchanged (A485-UN) peaks. For all other ChIP-seq and ATAC-seq experiments, clustering is performed based solely on the effect of A485 (Part 2). If the number of UP or DN peaks were less than 250 peaks, the peaks were excluded from the downstream analyses.

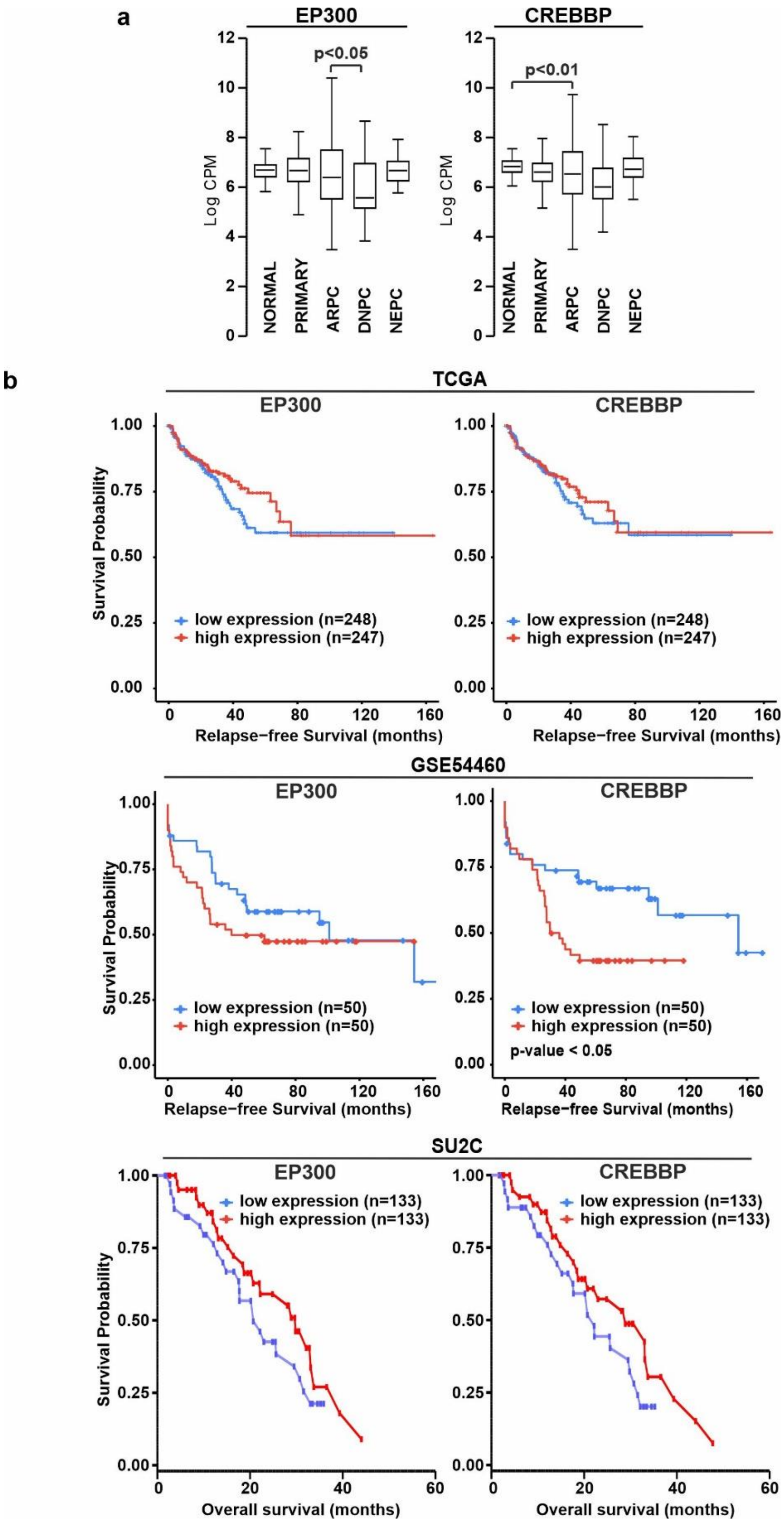

Supplementary Fig. S3. Analysis of *EP300/CREBBP* expression and survival from PCa patients. (**a**) Box plots represent the normalized log2 count per million (CPM) values of *EP300* and *CREBBP* expression in PCa patients (Prostate Cancer Atlas data). Data represent normal, primary cancer, AR-positive castration resistant prostate cancer (CRPC) (ANPC), double-negative CRPC (DNPC), and neuroendocrine CRPC (NEPC). The Statistical significance calculated using One-way ANOVA with Bonferroni *post hoc* test. (**b**) Relapse-free survival of three PCa patient datasets; TCGA (upper), GSE54460 (middle), and SU2C (lower), with high (red) or low (blue) expression levels of *EP300* (left) and *CREBBP* (right). Statistical significance calculated with log-rank test. The number of patients (n) in each group is displayed in the graph.

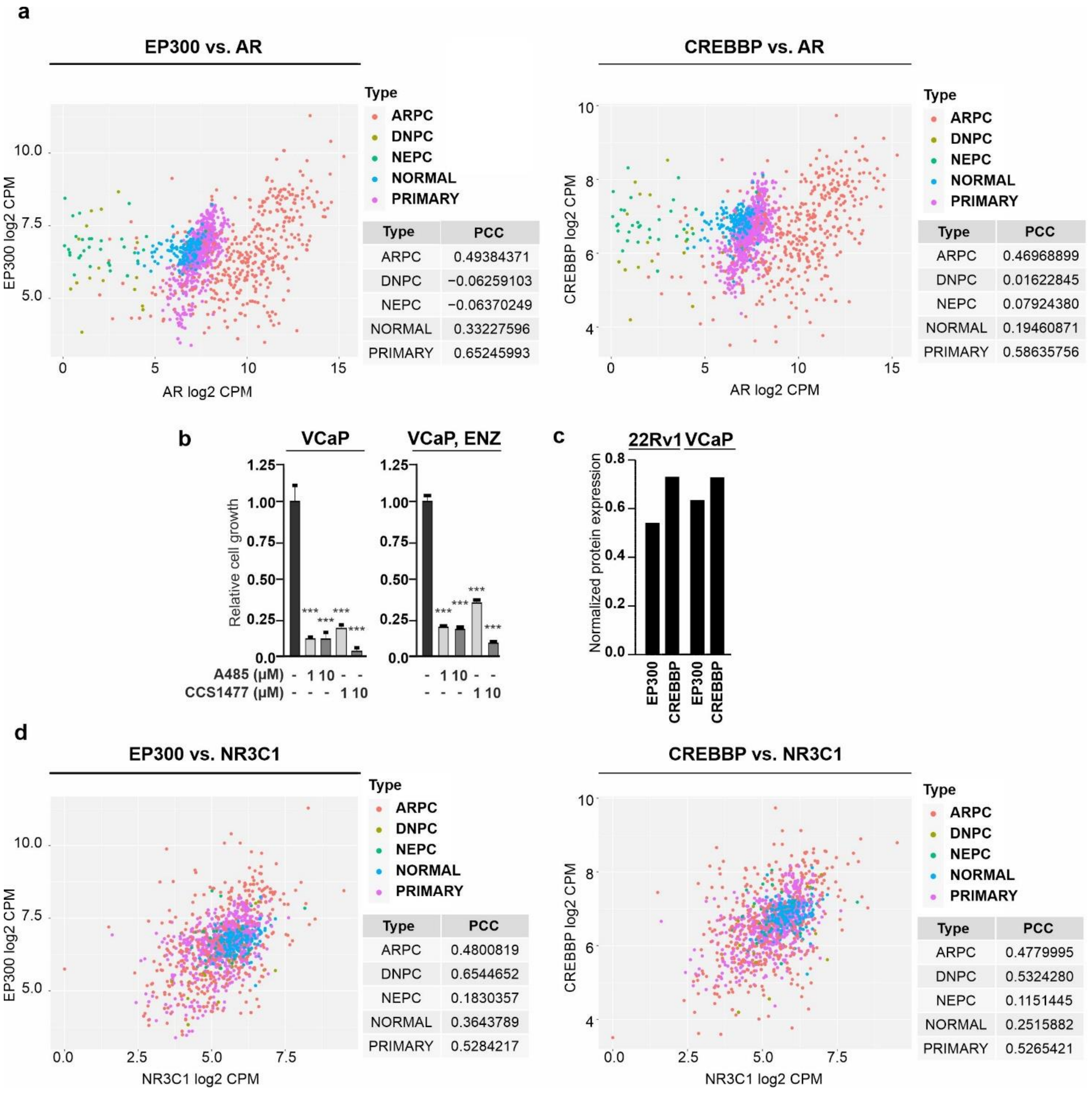

Supplementary Fig. S4. The expression correlation between EP300/CREBBP and steroid receptors. (a) Scatter plot of *EP300* (left) or *CREBBP* (right) (x-axis) and *AR* (y-axis) expression from RNA-seq data from Prostate Cancer Atlas patient datasets. Data represent normal, primary cancer, AR-positive castration resistant prostate cancer (CRPC) (ANPC), double-negative CRPC (DNPC), and neuroendocrine CRPC (NEPC). Correlation displayed as Pearson Correlation Coefficiency (PCC). **(b)** Bar graphs depict relative cell proliferation of ENZ-naïve (left) and ENZ-exposed (right) VCaP cells treated with indicated EP300/CREBBP inhibitors for 96 h. Data is normalized to cell proliferation at the start of the experiment, and represent mean±SD, n=4. Statistical significance calculated using One-way ANOVA with Bonferroni post hoc test. *, p<0.05; **, p<0.01; ***, p<0.001. **(c)** Bar graphs depict normalized quantitative protein levels for EP300 and CREBBP in 22Rv1 and VCaP cells. Quantitative proteomics data was obtained from *The Cancer Cell Line Encyclopedia* (CCLE). **(d)** Scatter plot of *EP300* (left) or *CREBBP* (right) (x-axis) and *NR3C1* (GR) (y-axis) expression from RNA-seq data from Prostate Cancer Atlas patient dataset. Data represent normal, primary cancer, ANPC, DNPC, and NEPC. Correlation displayed as PCC.

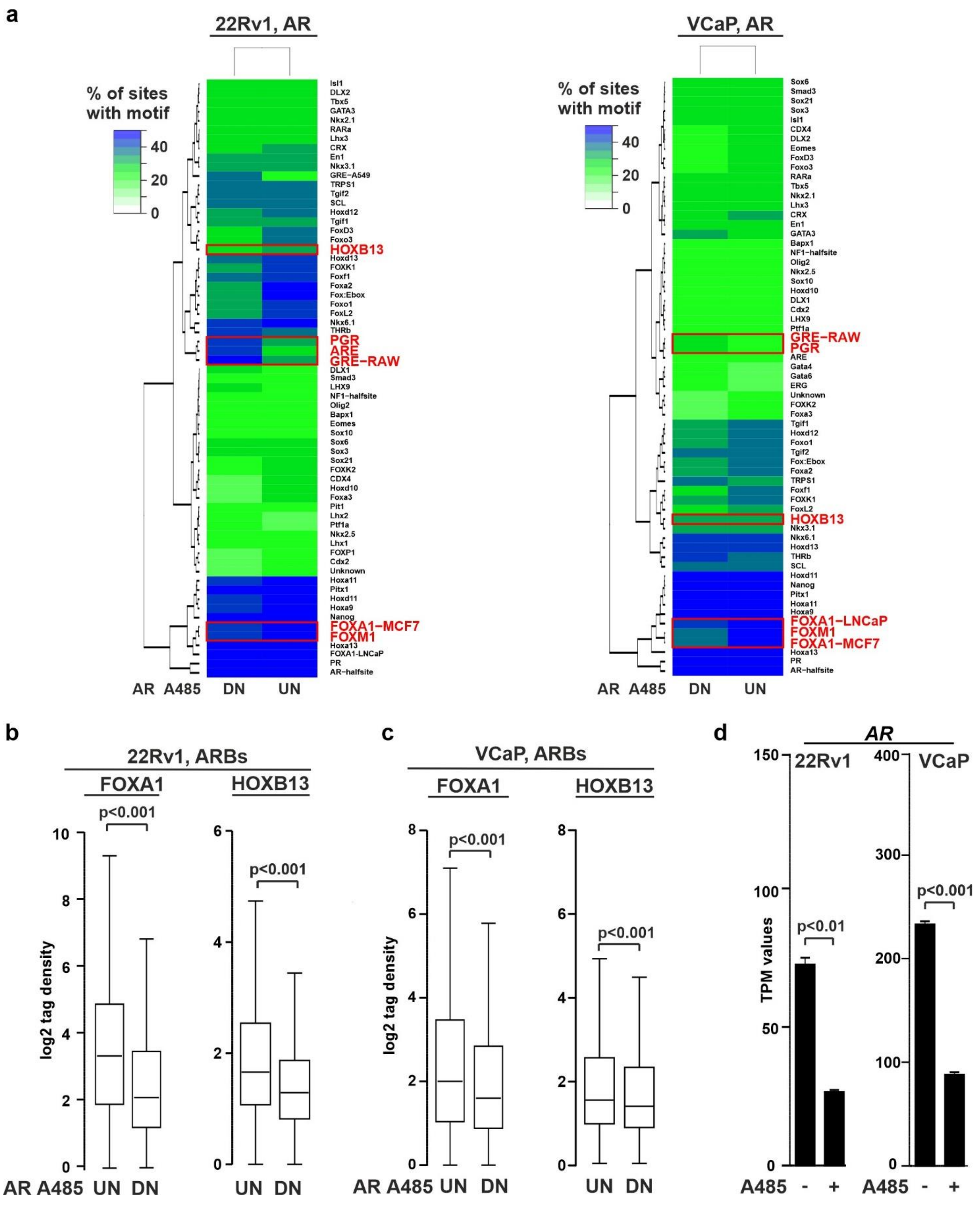

Supplementary Fig. S5. Enrichment of motifs at ARBs. (**a**) *De novo* motif enrichment at indicated ARBs in 22Rv1 (left) and VCaP (right) cells. Enrichment is displayed as a heatmap representing % of sites with motif. The scale is displayed on the side of the heatmap with white-green-blue indicating the prevalence of enrichment. Red rectangle highlights relevant motifs. (**b-c**) Box plots represent the normalized log2 tag density of FOXA1 ChIP-seq and HOXB13 ChIP-seq at indicated sites in (**b**) 22Rv1 cells and (**c**) VCaP cells. Statistical significance calculated using unpaired t-test. (**d**) Bar graphs depict *AR* expression levels in 22Rv1 and VCaP cells from RNA-seq data. Data shown as transcripts per million (TPM). Statistical significance calculated with unpaired t-test, n=2.

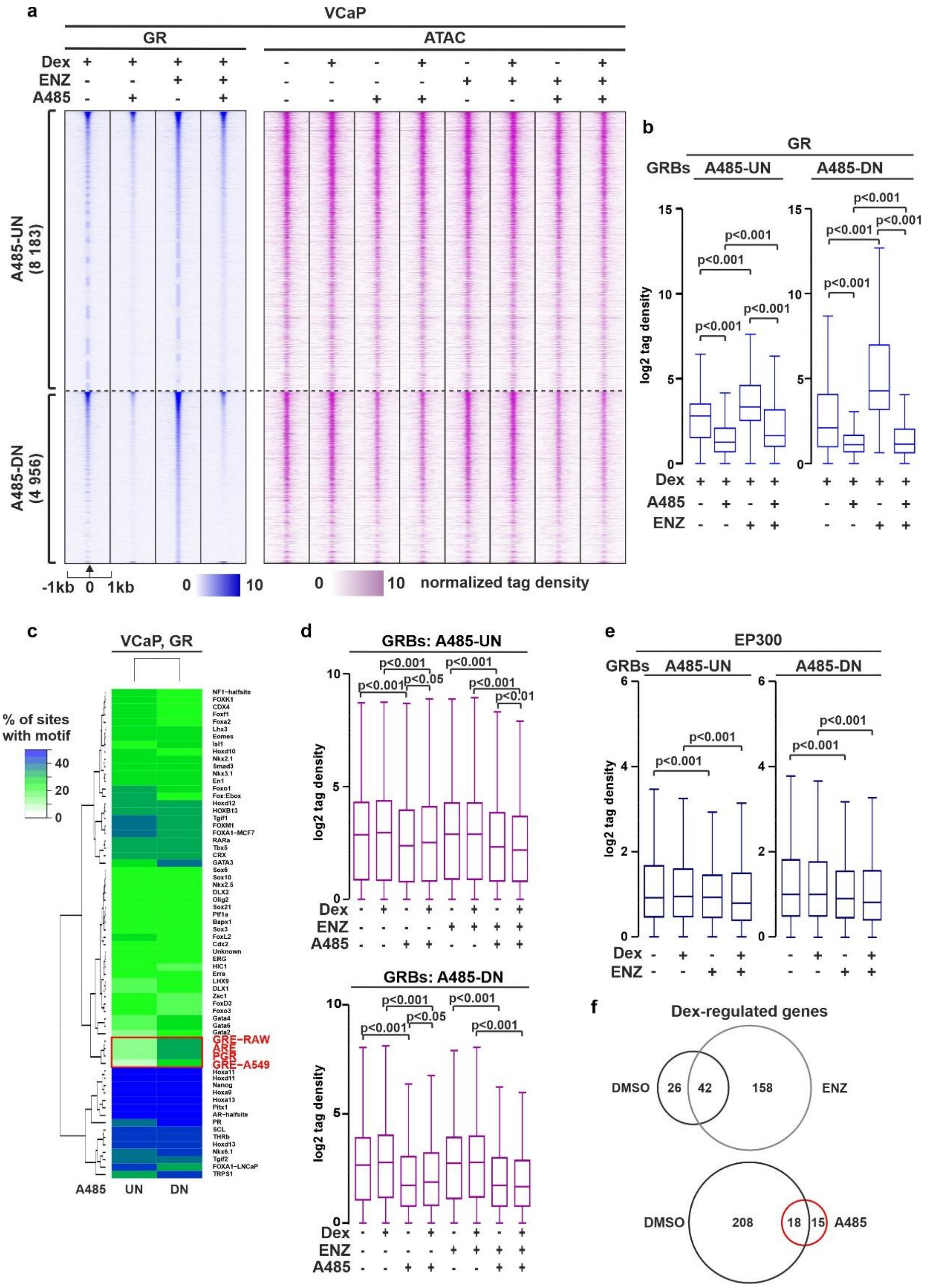

Supplementary Fig. S6. GR ChIP-seq and ATAC-seq analyses from VCaP cells upon A485 exposure. (**a**) GR ChIP-seq and ATAC-seq profiles at A485-UN and A485-DN sites in ENZ-naïve and ENZ-exposed VCaP cells. UN represents unchanged and DN decreased GRBs. Each heatmap represents ±1 kb around the center of the GR peak. Binding intensity (tags per bp per site) scale is noted below on a linear scale. **(b)** Box plots represent the normalized log2 tag density of GR ChIP-seq at indicated sites in VCaP cells. (**c**) *De novo* motif enrichment at indicated GRBs in VCaP cells. Enrichment is displayed as a heatmap representing % of sites with motif. The scale is displayed on the side of the heatmap with white-green-blue indicating the prevalence of enrichment. Red rectangle highlights relevant motifs. (**d**) Box plots represent the normalized log2 tag density of ATAC-seq at A485-UN (upper) and A485-DN (lower) sites in VCaP cells. (**e**) Box plots represent the normalized log2 tag density of EP300 ChIP-seq at indicated sites in VCaP cells. Statistical significance in box plots calculated using One-way ANOVA with Bonferroni *post hoc* test. (**f**) Venn diagrams of Dex-regulated genes from (upper) ENZ-naïve (black circle) and ENZ-exposed (grey circle) VCaP cells, and (lower) DMSO (black circle) and A485 (red circle) exposed VCaP cells.

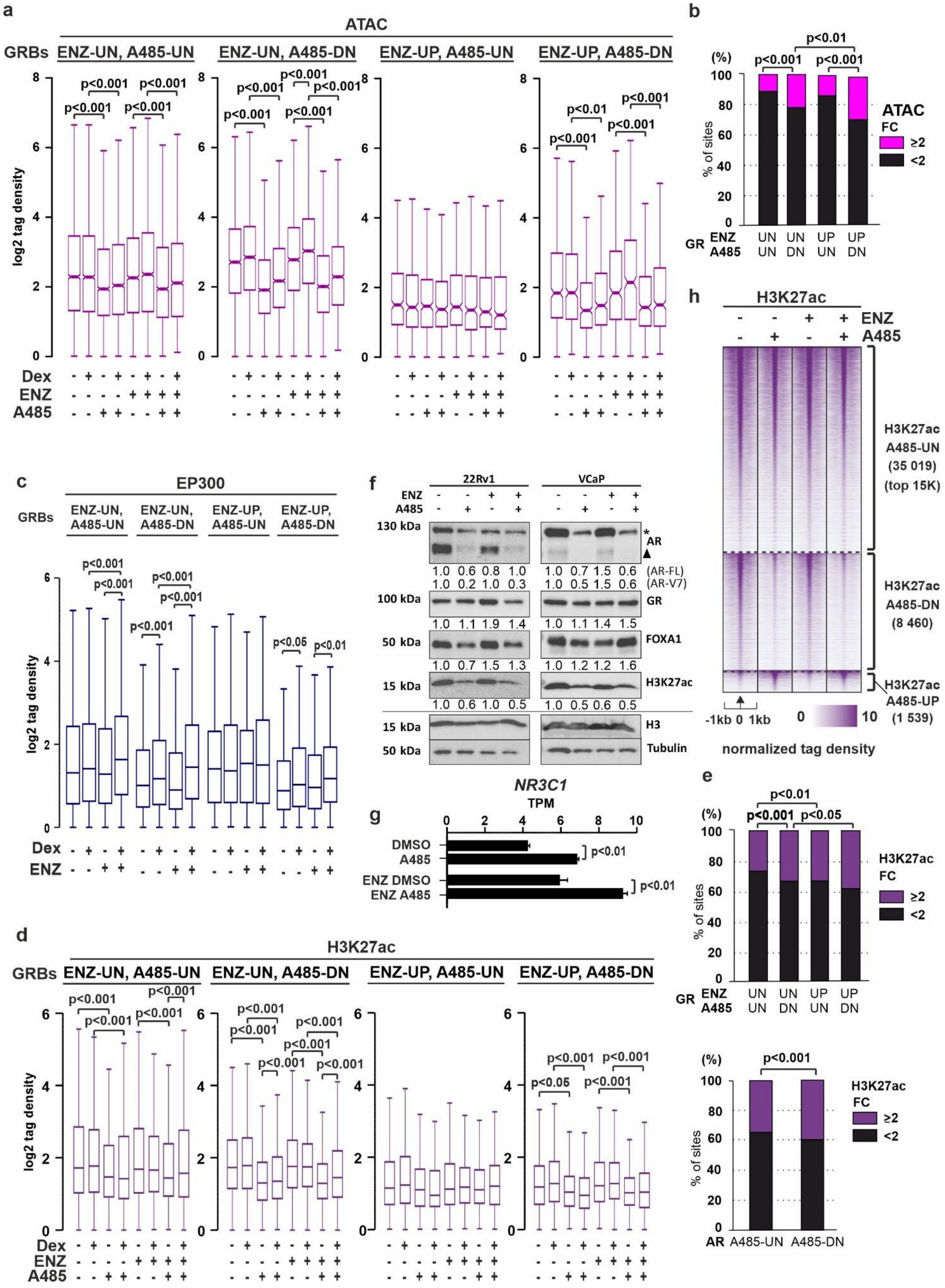

a
ATAC
GRBs ENZ-UN, A485-UN
ENZ-UN, A485-DN
ENZ-UP, A485-UN
ENZ-UP, A485-DN
log2 tag density
p<0.001
p<0.01
Dex
ENZ
A485
b
(%)
% of sites
ATAC
FC
≥2
<2
GR ENZ
A485
UN UN UP UP
UN DN UN DN
c
EP300
GRBs
ENZ-UN, A485-UN
ENZ-UN, A485-DN
ENZ-UP, A485-UN
ENZ-UP, A485-DN
p<0.05
f
22Rv1
VCaP
ENZ
A485
130 kDa
AR
(AR-FL)
(AR-V7)
100 kDa
GR
50 kDa
FOXA1
15 kDa
H3K27ac
H3
Tubulin
g
NR3C1
TPM
DMSO
A485
ENZ DMSO
ENZ A485
h
H3K27ac
H3K27ac A485-UN (35 019) (top 15K)
H3K27ac A485-DN (8 460)
H3K27ac A485-UP (1 539)
-1kb 0 1kb
normalized tag density
d
H3K27ac
e
H3K27ac
FC
AR A485-UN A485-DN

Supplementary Fig. S7. Analysis of chromatin accessibility, EP300 and H3K27ac enrichment at GRBs. (**a**) Box plots represent the normalized log2 tag density of ATAC-seq at ENZ-UN A485-UN, ENZ-UN A485-DN, ENZ-UP A485-UN and ENZ-UP A485-DN sites in 22Rv1 cells. Statistical significance calculated using One-way ANOVA with Bonferroni *post hoc* test. **(b)** Bar graphs depict the proportion of indicated GR binding clusters in 22Rv1 cells with less than (black) or more than (magenta) 2-fold reduction in ATAC-seq signal after A485 treatment. Statistical significance calculated using Chi-squared test. **(c-d)** Box plots represent the normalized log2 tag density of **(c)** EP300-ChIP-seq and **(d)** H3K27ac-ChIP-seq at ENZ-UN A485-UN, ENZ-UN A485-DN, ENZ-UP A485-UN and ENZ-UP A485-DN sites in 22Rv1 cells. Statistical significance calculated using One-way ANOVA with Bonferroni *post hoc* test. **(e)** Bar graphs depict the proportion of indicated GR (upper) or AR (lower) binding clusters in 22Rv1 cells with less than (black) or more than (purple) 2-fold reduction in H3K27ac ChIP-seq signal after A485 treatment. Statistical significance calculated using Chi-squared test. (**f**) Immunoblotting of GR, AR, FOXA1, H3K27ac, H3 and tubulin (TUB) protein levels in ENZ-naïve and ENZ-exposed 22Rv1 (left) and VCaP (right) cells treated with DMSO or A485. Quantification of AR (star=full length AR, triangle= AR-V7), GR, FOXA1, and H3K27ac levels is shown below the immunoblot. (**g**) Bar graphs depict *NR3C1* expression levels in 22Rv1 and VCaP cells from RNA-seq data. Data shown as transcripts per million (TPM). Statistical significance calculated with unpaired t-test, n=2. (**h**) H3K27ac ChIP-seq profile at H3K27ac A485-UN, H3K27ac A485-DN and H3K27ac A485-UP sites in ENZ-naïve and ENZ-exposed 22Rv1 cells. Heatmaps represents ±1 kb around the center of the H3K27ac peak. Binding intensity (tags per bp per site) scale is noted below on a linear scale.

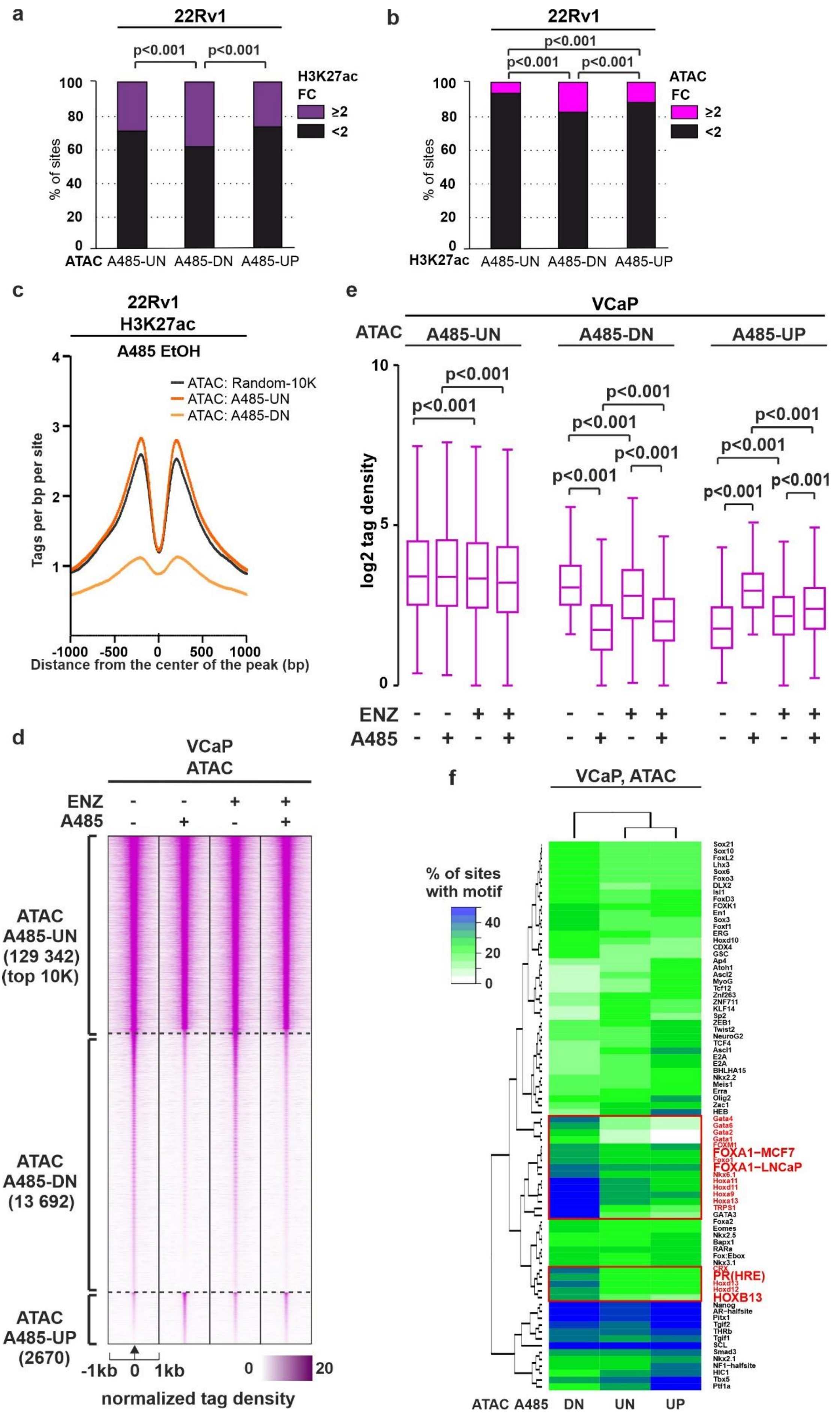
a
22Rv1
p<0.001
p<0.001
H3K27ac
FC
≥2
<2
% of sites
100
80
60
40
20
0
ATAC A485-UN A485-DN A485-UP
b
22Rv1
p<0.001
p<0.001
p<0.001
ATAC
FC
≥2
<2
% of sites
H3K27ac A485-UN A485-DN A485-UP
c
22Rv1
H3K27ac
A485 EtOH
ATAC: Random-10K
ATAC: A485-UN
ATAC: A485-DN
Tags per bp per site
-1000 -500 0 500 1000
Distance from the center of the peak (bp)
e
VCaP
ATAC
A485-UN
A485-DN
A485-UP
log2 tag density
p<0.001
ENZ
A485
d
VCaP
ATAC
ENZ
A485
ATAC A485-UN (129 342) (top 10K)
ATAC A485-DN (13 692)
ATAC A485-UP (2670)
-1kb 0 1kb
0
20
normalized tag density
f
VCaP, ATAC
% of sites with motif
40
20
0
Sox21
Sox10
FoxL2
Lhx3
Sox6
Foxo3
DLX2
Isl1
FoxD3
FOXK1
En1
Sox3
Foxf1
ERG
Hoxd10
CDX4
GSC
Ap4
Atoh1
Ascl2
MyoG
Tcf12
Znf263
ZNF711
KLF14
Sp2
ZEB1
Twist2
NeuroG2
TCF4
Ascl1
E2A
E2A
BHLHA15
Nkx2.2
Meis1
Erra
Olig2
Zac1
HEB
Gata4
Gata6
Gata2
Gata1
FOXM1
FOXA1-MCF7
Foxo1
FOXA1-LNCaP
Nkx6.1
Hoxa11
Hoxd11
Hoxa9
Hoxa13
TRPS1
GATA3
Foxa2
Eomes
Nkx2.5
Bapx1
RARa
Fox:Ebox
Nkx3.1
CRX
PR(HRE)
Hoxd13
Hoxd12
HOXB13
Nanog
AR-halfsite
Pitx1
Tgif2
THRb
Tgif1
SCL
Smad3
Nkx2.1
NF1-halfsite
HIC1
Tbx5
Ptf1a
ATAC A485 DN UN UP

Supplementary Fig. S8. Analysis of global chromatin accessibility in VCaP cells upon A485 exposure. (**a**) Bar graphs depict the proportion of indicated ATAC-seq clusters in 22Rv1 cells with less than (black) or more than (purple) 2-fold reduction in H3K27ac ChIP-seq signal after A485 treatment. Statistical significance calculated using Chi-squared test. (**b**) Bar graphs depict the proportion of indicated H3K27ac enriched clusters in 22Rv1 cells with less than (black) or more than (magenta) 2-fold reduction in ATAC-seq signal after A485 treatment. Statistical significance calculated using Chi-squared test. (**c**) Aggregate plot represents the binding intensity (tags per bp per site) of A485 treated H3K27ac ChIP-seq samples at indicated ATAC sites in ENZ-naïve 22Rv1 cells. Random 10 000 ATAC sites are randomly selected from the union of all ATAC sites. Aggregate plot represents ±1 kb around the center of the ATAC peak and it is normalized to a total of 10 million reads. (**d**) ATAC-seq profile at ATAC A485-UN, ATAC A485-DN and ATAC A485-UP sites in ENZ-naïve and ENZ-exposed VCaP cells. UN represents unchanged, UP increased and DN decreased open chromatin sites. Each heatmap represents ±1 kb around the center of the ATAC peak. Binding intensity (tags per bp per site) scale is noted below on a linear scale. (**e**) Box plots represent the normalized log2 tag density of ATAC-seq at indicated sites. Statistical significance calculated using One-way ANOVA with Bonferroni *post hoc* test. (**f**) *De novo* motif enrichment at indicated open chromatin sites in VCaP cells. Enrichment is displayed as a heatmap representing % of sites with motif. The scale is displayed on the side of the heatmap with white-green-blue indicating the prevalence of enrichment. Red rectangle highlights relevant motifs.

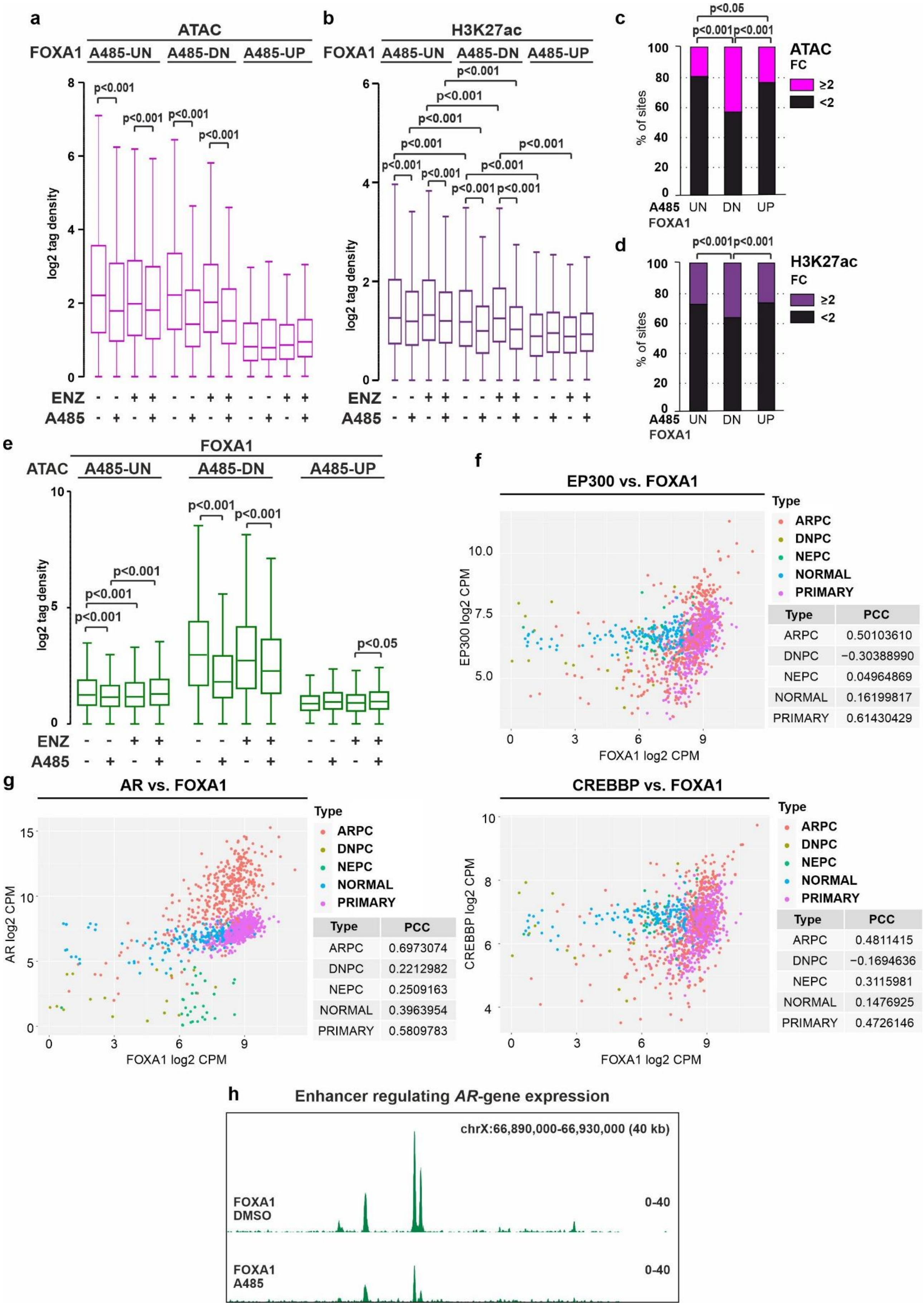
a
ATAC
FOXA1 A485-UN A485-DN A485-UP
p<0.001
p<0.001
p<0.001
p<0.001
log2 tag density
ENZ
A485
b
H3K27ac
FOXA1 A485-UN A485-DN A485-UP
p<0.001
p<0.05
log2 tag density
ENZ
A485
c
ATAC
FC
≥2
<2
% of sites
A485 UN DN UP
FOXA1
d
H3K27ac
FC
≥2
<2
% of sites
A485 UN DN UP
FOXA1
e
FOXA1
ATAC A485-UN A485-DN A485-UP
log2 tag density
ENZ
A485
f
EP300 vs. FOXA1
EP300 log2 CPM
FOXA1 log2 CPM
Type
ARPC
DNPC
NEPC
NORMAL
PRIMARY
Type PCC
ARPC 0.50103610
DNPC −0.30388990
NEPC 0.04964869
NORMAL 0.16199817
PRIMARY 0.61430429
g
AR vs. FOXA1
AR log2 CPM
FOXA1 log2 CPM
Type PCC
ARPC 0.6973074
DNPC 0.2212982
NEPC 0.2509163
NORMAL 0.3963954
PRIMARY 0.5809783
CREBBP vs. FOXA1
CREBBP log2 CPM
FOXA1 log2 CPM
Type PCC
ARPC 0.4811415
DNPC −0.1694636
NEPC 0.3115981
NORMAL 0.1476925
PRIMARY 0.4726146
h
Enhancer regulating AR-gene expression
chrX:66,890,000-66,930,000 (40 kb)
FOXA1 DMSO
0-40
FOXA1 A485
0-40

Supplementary Fig. S9. Enrichment of H3K27ac and chromatin accessibility at FOXA1 binding sites. (**a-b**) Box plots represent the normalized log2 tag density of (**a**) ATAC-seq and (**b**) H3K27ac ChIP-seq at FOXA1 A485-UN, FOXA1 A485-DN and FOXA1 A485-UP sites. (**c-d**) Bar graphs depict the proportion of indicated FOXA1 binding clusters in 22Rv1 cells with (**c**) less than (black) or more than (magenta) 2-fold reduction in ATAC-seq signal, or (**d**) less than (black) or more than (purple) 2-fold reduction in H3K27ac ChIP-seq signal after A485 treatment (DMSO/A485). Statistical significance calculated using Chi-squared test. **(e)** Box plots represent the normalized log2 tag density of FOXA1 ChIP-seq at ATAC A485-UN, ATAC A485-DN and ATAC A485-UP sites in 22Rv1 cells. Statistical significance in box plots calculated using One-way ANOVA with Bonferroni *post hoc* test. (**f**) Scatter plot of *EP300* (upper) or *CREBBP* (lower) (x-axis) and *FOXA1* (y-axis) expression from RNA-seq data from Prostate Cancer Atlas patient dataset. (**g**) Scatter plot of *AR* (x-axis) and *FOXA1* (y-axis) expression from RNA-seq data from Prostate Cancer Atlas patient dataset. Data represent normal, primary cancer, AR-positive castration resistant prostate cancer (CRPC) (ANPC), double-negative CRPC (DNPC), and neuroendocrine CRPC (NEPC). Correlation displayed as Pearson Correlation Coefficiency (PCC). (**h**) Genome browser track example of FOXA1 ChIP-seq at enhancer regulating *AR*-gene expression in 22Rv1 cells.

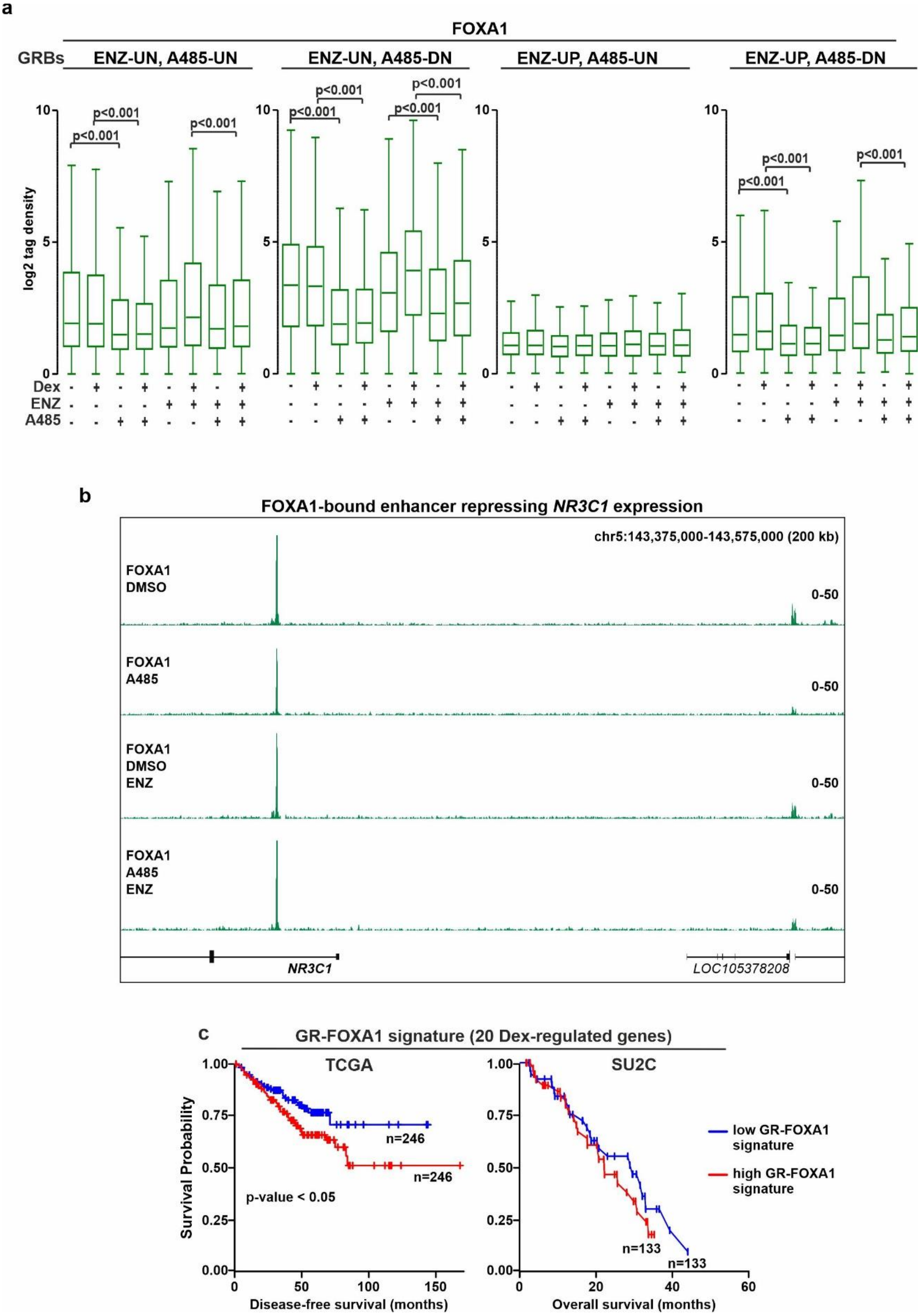

Supplementary Fig. S10. Enrichment of FOXA1 occupancy at GRBs. (**a**) Box plots represent the normalized log2 tag density of FOXA1 ChIP-seq at ENZ-UN A485-UN, ENZ-UN A485-DN, ENZ-UP A485-UN and ENZ-UP A485-DN sites in 22Rv1 cells. Statistical significance calculated using One-way ANOVA with Bonferroni *post hoc* test. (**b**) Genome browser track examples of FOXA1 ChIP-seq at enhancer repressing *NR3C1* expression in 22Rv1 cells. (**c**) Disease-free survival of patients with high (red) or low (blue) levels of GR-FOXA1 signature (20 Dex-regulated genes) in TCGA (left) or SU2C (right) datasets. Statistical significance calculated with log-rank test.

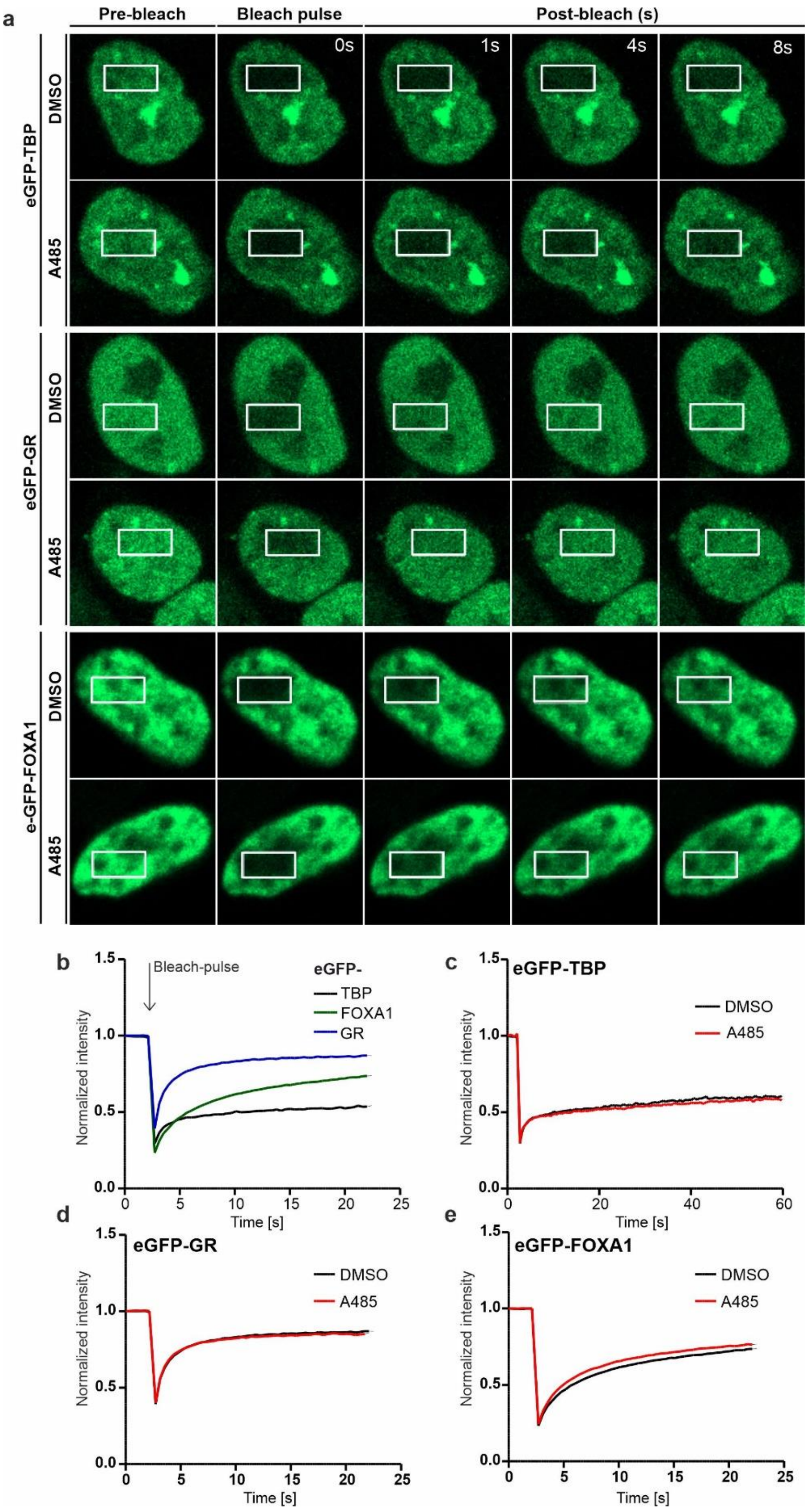

Supplementary Fig. S11. A485 influences the nuclear mobility of FOXA1. (**a**) Fluorescence recovery after photobleaching (FRAP) analyses were conducted in 22Rv1 cells transfected with eGFP-tagged GR or FOXA1, as well as with TBP as control. Representative images of cell nuclei before bleaching (Pre-bleach), immediately after the bleach pulse (Bleach pulse), and during recovery (Post-bleach: 1 s, 4 s, and 8 s) are shown. (**b**) Line graph represents normalized recovery curves of eGFP-TBP (black), -FOXA1 (green) and -GR (blue) without A485 treatment. (**c-e**) Line graphs represent FRAP recovery curves of eGFP-TBP (**c**), -GR (**d**) and -FOXA1 (**e**) in DMSO (black) and A485 (red) treated samples. The number of single cells analyzed: GR, n = 75; FOXA1, n = 75; TBP, n = 12.

Description of Supplementary Tables

Supplementary Table S1. RNA-seq data. (Separate file)

Supplementary Table S2. Pathway enrichment analyses. (Separate file)

Supplementary Table S3. Public datasets utilized in the study. (Separate file)

Supplementary Table S4. ChIP-seq peaks and ATAC-seq tag distribution per position. (Separate file)

Supplementary Table S5. Motif enrichment analyses. (Separate file)

Supplementary Table S6. GR-FOXA1 signature genes.

**Accession number**	**Symbol**
NM_001363644	TBCEL
NM_001281429	PCED1B
NM_004613	TGM2
NR_104213	ANGPTL4
NM_001300766	TSEN15
NM_033102	SLC45A3
NM_014899	RHOBTB3
NM_000240	MAOA
NM_153023	SPATA13
NM_020177	FEM1C
NM_018948	ERRFI1
NM_001322838	KCNMA1
NM_001347706	CHRNA2
NR_103485	PLPP1
NM_001243281	ALCAM
NM_019018	OTULINL
NM_001256793	BMPR1B
NM_001320986	IL1R1
NM_001134194	ACPP
NM_018018	SLC38A4
